# Dysregulation of Immature Sertoli Cell Functions by Exposure to Acetaminophen and Genistein in Rodent Cell Models

**DOI:** 10.3390/cells12131804

**Published:** 2023-07-07

**Authors:** Maia Corpuz-Hilsabeck, Nicole Mohajer, Martine Culty

**Affiliations:** Department of Pharmacology and Pharmaceutical Sciences, Alfred E. Mann School of Pharmacy and Pharmaceutical Sciences, University of Southern California, Los Angeles, CA 90089, USA

**Keywords:** Sertoli cell, genistein, acetaminophen, proliferation, gene expression, Sox9, cyclooxygenases, functional pathways

## Abstract

Sertoli cells are essential for germ cell development and function. Their disruption by endocrine disrupting chemicals (EDCs) or drugs could jeopardize spermatogenesis, contributing to male infertility. Perinatal exposure to EDCs and acetaminophen (APAP) disrupts male reproductive functions in animals and humans. Infants can be exposed simultaneously to the dietary soy phytoestrogen genistein (GEN) and APAP used for fever or pain relief. Our goal was to determine the effects of 10–100 µM APAP and GEN, alone or mixed, on immature Sertoli cells using mouse TM4 Sertoli cell line and postnatal-day 8 rat Sertoli cells, by measuring cell viability, proliferation, prostaglandins, genes and protein expression, and functional pathways. A value of 50 µM APAP decreased the viability, while 100 µM APAP and GEN decreased the proliferation. Sertoli cell and eicosanoid pathway genes were affected by GEN and mixtures, with downregulation of Sox9, *Cox1*, *Cox2*, and genes relevant for Sertoli cell function, while genes involved in inflammation were increased. RNA-seq analysis identified p53 and TNF signaling pathways as common targets of GEN and GEN mixture in both cell types. These results suggest that APAP and GEN dysregulate immature Sertoli cell function and may aid in elucidating novel EDC and drug targets contributing to the etiology of male infertility.

## 1. Introduction

Sertoli cells (SCs), the “nurse cells” of germ cells in the male gonad [1], play an essential role in the organization of seminiferous cords, testis-specific vascular patterning, the appearance of other somatic cell types such as Leydig and peritubular myoid cells in testis development, and the regulation of spermatogenesis throughout life [2,3]. Androgen receptor expression delineates their switch from an immature-proliferating status to the mature, androgen responsive adult-type Sertoli cells formed at pre-puberty [1,3]. Fetal and/or perinatal exposures to environmental endocrine disrupting chemicals (EDCs) during male gonad development can have long-lasting effects thereafter, including disorders such as cryptorchidism, Sertoli-cell-only syndrome, and infertility [4,5].

Phthalates (phthalic acid esters) are a class of EDCs found ubiquitously in the environment that cause male reproductive disorders [4,6]. Di(2-ethylhexyl) phthalate (DEHP) and its biologically active metabolite mono(2-ehtylhexyl) phthalate (MEHP) are found to be commonly used in shampoo, cosmetics, hairspray, food packaging, and medical equipment. Xenoestrogens are also common in the environment. Among them, the phytoestrogen genistein (GEN) is found mainly in soy-based food products, and humans are exposed to it through diet; babies fed soy-based baby formula are particularly exposed to high levels of GEN [7,8]. In utero exposure to GEN was shown to alter the testicular function and signaling pathways in neonatal and adult rats [9,10]. There are limited studies examining the effects of EDC mixtures on the male reproductive system and their potential contribution to reproductive disorders, including male infertility. In previous in vivo studies, we showed that in utero exposure to mixtures of GEN and DEHP at doses found in humans dysregulated more male reproductive development than individual EDCs. Specifically, a higher rate of abnormal testicular phenotypes consisting of atrophied tubules with a Sertoli-cell-only phenotype was observed in postnatal day 120 (PND120) rat testes exposed in utero to 0.1 and 10 mg/kg/day of GEN + DEHP mixtures [10,11,12]. These studies unveiled short-term oxidative stress events and long-term changes in innate immune cells, particularly macrophages, suggesting inflammatory processes. Genes and proteins representative of Leydig cells, germ cells, and Sertoli cells were affected by in utero exposures to GEN and DEHP. The expression of the Sertoli cell markers *Abp* and *Amh* was downregulated in PND6 and PND120 rat testes following in utero exposures to GEN and DEHP mixtures [10,11,12]. The in vitro study of the effects of GEN and MEHP at 10 µM and up on the C18-4 spermatogonial cell line further confirmed that undifferentiated spermatogonia were direct targets of these EDCs, and showed that the eicosanoid pathway was disrupted, including the gene and protein expression of Cox1 and Cox2 and prostaglandins production [13]. These data put together, drive a compelling argument that exposure to EDCs and drugs with Cox inhibiting properties such as APAP and NSAIDs during perinatal phases of male gonad development could also disrupt immature Sertoli cell function and development.

The pharmaceutical class of non-steroidal anti-inflammatory drugs (NSAIDs) such as ibuprofen (Ibu) and analgesic drugs such as acetaminophen (N-acetyl-para-aminophenol; APAP; paracetamol) are commonly used to treat infants diagnosed with pain, severe fever, and some chronic diseases [14,15]. The global prevalence of analgesic use across 10,000 pregnant women in the U.S. in 2005 and 6500 pregnant women in 2013 showed that acetaminophen was the highest used analgesic compared to NSAIDs or other analgesic drugs [16]. These pharmaceutical drugs are increasingly scrutinized for their effects on reproductive development and function [15,17]. Intergenerational effects of high exposure to APAP and IB during pregnancy are suspected to be at the origin of second and third-generation offspring experiencing lowered sperm count, delayed Sertoli cell maturation, and a decrease in spermatogonia A pool size [18].

The overall goal of the present study was to examine the effects of APAP/NSAIDs and EDCs on immature Sertoli cells, to simulate infant exposure to these chemicals, individually or as mixtures, as it can happen in the clinic setting or at home. There is a gap in knowledge on the possible contribution of Sertoli cell dysregulation by EDCs and pharmaceuticals on male fertility. While some studies reported the involvement of Sertoli cells in adverse responses of the reproductive system to EDCs such as cadmium, phthalates, BPA and analogs, pesticides, and fungicides, none of them studied the effects of genistein and APAP alone or mixed on immature Sertoli cell functions, to the best of our knowledge. Studies on APAP in a mixture with estrogenic and anti-androgenic EDCs found in the environment showed that these exposures disrupted male fertility [3,19], in support of our hypothesis. We aimed at finding whether mixtures of EDCs and APAP/NSAIDs to which infants are commonly exposed could worsen the effects of individual EDCs and/or pharmaceuticals in immature Sertoli cells. The expectation was that exposure to these chemicals would dysregulate immature Sertoli cell function and development, suggesting potential long-term effects that could contribute to male infertility and reproductive disorders. 

## 2. Materials and Methods

### 2.1. Chemicals

Genistein (4′,5,7-Trihydroxyisoflavone) (GEN, G), mono (2-ethylhexyl) phthalate (MEHP, M), acetaminophen (APAP, A), and Ibuprofen (Ibu, Ib) were purchased from Sigma-Aldrich (St. Louis, MO, USA). Stock solution of 10^−2^ M GEN was dissolved in ultra-pure grade dimethyl sulfoxide (DMSO from VWR International, Radnor, PA, USA). An amount of 10^−2^ M stock solutions of MEHP, APAP, and IB were dissolved in 100% ethanol (Gold Shield, Hayward, CA, USA). Stock solutions were stored at −20 °C.

### 2.2. TM4 Mouse Sertoli Cell Line Culture

Immature murine Sertoli TM4 cell line (Cat. no. CRL-1715, ATCC, Manassas, VA, USA) was cultured in Gibco™ DMEM containing 4.5 g/L d-Glucose, L-glutamine, and 110 mg/L of sodium pyruvate (Thermo Fisher Scientific, Waltham, MA, USA) supplemented with either 10% heat-inactivated Regular FBS (REG-FBS) or charcoal-stripped FBS (CS-FBS) (Sigma Aldrich, St. Louis, MO, USA) and 1% Penicillin-Streptomycin (Corning™). After reaching adequate confluence for treatment plating, Sertoli cells were washed twice with PBS, lifted by short trypsin treatment, and plated in a medium containing either 10% Regular (Reg) or 10% Charcoal-stripped (CS) FBS (Sigma-Aldrich, St. Louis, MO, USA) in culture plates as needed for treatments. Cells were grown overnight to reach 60–70% confluency in media containing either REG-FBS or CS-FBS, then treated accordingly. 

### 2.3. Primary Immature Rat Sertoli Cell Isolation

Rat immature Sertoli cells were isolated from postnatal day (PND) 8 rat testes as previously described [20,21,22] (Supplementary Figure S1A). Briefly, testes from 10 rats/experiment were collected and decapsulated, then tissue dissociation was performed by sequential enzymatic dissociation. Interstitial cells were first dissociated using 2 mg/mL type IV collagenase mixed with 10 mg/mL hyaluronidase and 1 mg/mL DNAse I (all from Sigma Aldrich) in RPMI 1640 medium (Invitrogen, Thermo Fisher Scientific, Waltham, MA, USA) +2% Penicillin-Streptomycin and 1% Amphotericin B (fungicide) for 30 min in a shaking water bath at 37 °C. The seminiferous tubules were pelleted by sedimentation and the supernatnat containing interstitial cells was discarded. Seminiferous tubules were then dissociated with 0.25% trypsin-1 mM EDTA and DNAse I for 15 min in a shaking water bath at 37 °C. After mechanical dissociation by flushing in pipet and a brief sedimentation, the cell suspension was collected. If a large pellet was visible after sedimentation, a 2nd short trypsination could be applied to digest the remaining tubules. Cell suspensions were rapidly diluted at 1:1 with RPMI 1640 + 10% Reg-FBS to inactivate trypsin, followed by filtration to remove fragments, 5 min of centrifugation at 800× *g*, and the resuspension of cell pellet containing Sertoli, myoid and germ cells, in RPMI 1640 with antibiotics and 5% CS-FBS. Next, overnight differential plating was done at 37 °C, 3.5% CO_2_, to allow Sertoli cells to attach to culture dishes while germ cells remained floating. Floating cells (mainly germ cells) were removed the next morning. Adherent cell layers (mainly Sertoli cells) were then switched to DMEM with antibiotics and 10% CS-FBS, the same medium as used for TM4 cell cultures, and incubated for 8 h. PND8 rat Sertoli cells were then washed twice with PBS, lifted by short trypsin treatment, centrifuged, resuspended in medium with 10% CS-FBS, counted with hemocytometer (50–100 millions cells/cell preparation), diluted and plated in wells, and cultured at 37 °C, 3.5% CO_2_, as needed for treatments. Cells were kept for 5 h before adding treatments. 

Some PND8 rat Sertoli cells were plated in 8-well culture chambers (40,000 to 75,000/well) to assess PND8 Sertoli cell purity based on Vimentin IF staining for Sertoli cells, α-SMA IF staining for peritubular myoid cells, and nuclear DAPI dye for total cell numbers. Analysis with ImageJ versions 1.53k and 1.54d software of several samples from different experiments gave an average Sertoli cell purity of 72 ± 7% (Supplementary Figure S1B).

### 2.4. Sertoli Cell Treatments

Sertoli cells were plated in 6, 12, 24, or 96-well Corning culture-treated plates at various cell numbers depending on the assays performed, as indicated in the method sections below, using overall 2 to 3 times less TM4 cells than PND8 Sertoli cells as the primary cells grew slower in culture than the cell line. In both cases, cells were used at similar confluency in wells when treatments were added, and they generated adequate amounts of total RNA for mRNA analyses and fixed cell layers for IF analysis. All treatments were diluted in cell culture medium containing 10% of either REG-FBS or CS-FBS and filtered with 0.2 µm filters to prevent bacterial contamination. Cells were treated for 24 to 72 h with a vehicle or 10, 50, and 100 µM of APAP, IB, GEN, and MEHP alone or as mixtures, prepared in DMEM with 10% CS-FBS. The concentrations were selected to closely match the levels of the agents measured in human blood, as explained in previous studies [23,24,25,26]. All treatments were adjusted to contain the same final amount of DMSO and ethanol, each at 0.001% in the media—vehicle and treatments—that were added to cells.

### 2.5. Cell Viability/Proliferation with MTT Assay

The MTT assay, based on the measurement of the formazan formation by the reduction of tetrazolium MTT by mitochondrial dehydrogenase enzymes, is commonly used to assess both cell viability and proliferation. Sertoli cells were plated in 96-well Corning™ culture-treated microplates at 10,000 cells/well for TM4 cells and 20,000 cells/well for PND8 Sertoli cells, as well as incubated overnight at 37 °C 5% CO_2_. The MTT cell viability assay was followed according to the manufacturer’s protocol (Roche Cell proliferation kit I MTT, Sigma Aldrich, St. Louis, MO, USA). TM4 cells were cultured in DMEM supplemented with 10% heat-inactivated FBS exposed to APAP, IB, GEN, and MEHP alone or as a mixture over a 24-h period. MTT reagent was added at the end of 24-h incubation to incubate for an additional 4 h at 37 °C. Thereafter, 100 µL of solubilization solution was added to each well and incubated overnight at 37 °C with humidity. Conversion of MTT reagent to formazan crystals was measured using the VICTOR™ X5 Multilabel Plate Reader (PerkinElmer, Inc., Waltham, MA, USA). Data are expressed as a fold-change compared to the vehicle and calculated from both three experiments (TM4 cells) and two experiments (PND8 cells) conducted in triplicates.

### 2.6. Cell Proliferation with EdU Assay

Incorporation of EdU (5′-ethynyl-2′-deoxyuridine) in replicating DNA using the Click-iT™ EdU Alexa Fluor^TM^ 488 HCS assay was also used to measure TM4 cell proliferation. TM4 cells were plated in 96-well Corning™ culture-treated microplates at 10,000 cells/well and incubated overnight at 37 °C 5% CO_2_. Cells were treated for 24 h with EDCs and APAP/NSAIDs alone or as mixtures diluted in 10% CS-FBS supplemented medium. Incubation of 10 µM EdU (5′-ethynyl-2′-deoxyuridine) was performed over the last 6 h of the 24-h treatment as recommended by the Click-iT™ EdU HCS assay (Invitrogen, Carlsbad, CA, USA) manufacturer protocol. Cells in the culture plate were washed with 1× PBS and fixed to the culture plate using 4% paraformaldehyde followed by a 0.1% Triton X-100 permeabilization surfactant. Click-IT reaction was added to each well and incubated in the dark for 30 min at room temperature. Cells were subjected to a PBS wash step and 100 µL of HCS NuclearMask was added at a 1:2000 dilution per well. After a 30 min incubation at room temperature in the dark, cells were washed twice with PBS before imaging. Fluorescent-labeled DNA was measured by Biotek Cytation 5 imaging and quantification with GEN5 version 2.0 software (Biotek, Winooski, VT, USA).

### 2.7. Gene Expression Measured by qRT-PCR

TM4 Sertoli cells were plated at 100,000–150,000 cells/well, whereas primary PND8 rat Sertoli cells were plated 250,000–300,000 cells/well in 24-well culture plates. Zymo™ Quick-RNA Miniprep plus kit was used for total RNA extraction from TM4 cells and PND8 rat Sertoli cells (Zymo, Irvine, CA, USA). cDNA synthesis of TM4 cells and PND8 rat Sertoli cells from total RNA were extracted using the PrimeScript™ RT Master Mix (Takara Bio, Mountain View, CA, USA). The qPCR thermal cycler used for gene expression analysis was the BioRad CFX384 Touch Real-Time PCR System. Cycling conditions for qPCR were as follows: initial step at 95 °C followed by 40 cycles at 95 °C for 15 s, then 60 °C for 1 min. This was followed by both melting curves and cooling cycles. The SYBR Green system was used for gene amplification and the comparative threshold cycle (Ct) method used to analyze data, normalized to Gapdh (Table 1). 

### 2.8. PGD2 & PGE2 ELISA

TM4 cells plated at 250,000 cells/well in 12-well culture-treated plates and incubated overnight at 37 °C, 5% CO_2_. TM4 cells were treated for 24-h with either APAP, GEN, or the APAP + GEN mixture, all at 50 µM, dissolved in the culture medium containing 10% CS-FBS. In a previous study, we had verified that the DMEM medium containing CS-FBS contained less PGD2 and PGE2 than the medium supplemented with REG-FBS [13]. At the end of treatment, the cell supernatants (conditioned media) containing secreted PGs were collected and frozen until performing the ELISA assays for PGD2 and PGE2, using ELISA kits from Cayman Chemical (Ann Arbor, MI, USA) following the manufacturer protocol. Aliquots of medium supplemented with 10% CS-FBS from the same experiments were frozen for further use in the ELISA plates to determine the background level of PGs in the absence of cells. Conditioned media from control and treated TM4 Sertoli cells were also stored in −80 °C for later use in the ELISA assay. Measurement of %B/B0 was performed using a Cayman Chemical Excel spreadsheet for ELISA (Competitive) Analysis available online. Each condition was performed in two separate experiments with duplicates (*n* = 4).

### 2.9. Immunofluorescent (IF) Staining

TM4 cells or PND8 Sertoli cells were plated at either 125,000 cells/well in a 24-well culture-treated plate or 50,000 cells/well in 8-well chamber and grown overnight at 37 °C, 5% CO_2_. Treatment for 24 h followed thereafter. Cells were washed with 1× PBS and fixed to the culture plate or slide chamber with 4% paraformaldehyde. Cell were permeabilized by the addition of 0.1% Triton-X 100 in 1× PBS solution for a 10-min incubation at room temperature. The blocking step for IF staining was performed by adding 5% donkey serum in 0.5% BSA in 1× PBS solution to incubate for 30 min at room temperature. Antibodies incubated on cells at 1:100–1:300 diluted in 5% donkey serum in 0.5% BSA in 1× PBS solution overnight at 4 °C. Sox9 (anti-rabbit, catalog no. Ab185966, Abcam, Boston, MA, USA) COX1 (anti-rabbit, catalog no. 4841S, Cell Signaling, Danvers, MA, USA), COX2 (anti-rabbit, catalog no. ab52237, Abcam, Boston, MA, USA), ER-α (anti-rabbit, catalog no. MA1-310, Thermo Fisher Scientific, Waltham, MA, USA), PCNA (anti-mouse, catalog no. sc-56, Santa Cruz, Santa Cruz, CA, USA), and α-Tubulin (anti-mouse, catalog no. T9026, Thermo Fisher Scientific, Waltham, MA, USA) were used for IF staining in TM4 cells. The next day, the cell culture plate or slide chamber was washed three times with 1× PBS and decanting between washes at room temperature. A secondary antibody was used at 1:400 dilution in 5% donkey serum in 0.5% BSA in 1× PBS solution in which cells were incubated in the dark for 30 min at room temperature. Fluorescent-labeled cells were washed three times with 1× PBS to remove excess antibody solution and the slide chamber was removed before the addition of DAPI-mounting medium and a coverslip for glass slides. Cells were imaged and fluorescence quantified using the Biotek Cytation 5 imager and GEN5 software (Biotek, Winooski, VT, USA). Fold-change of immunofluorescent protein expression was compared between treatment and vehicle conditions (*n* = 4).

### 2.10. Whole Transcriptome Sequencing (Total RNA-Seq)

Whole transcriptome sequencing or total RNA-seq was performed on an Illumina Nextseq2000 platform by Keck Molecular Genomics Core (MGC) at the University of Southern California. Total RNA from both TM4 cells and 10 PND8 rat pups treated with vehicle or 50 µM APAP (A50), GEN (G50) or APAP + GEN (AG50) mixture was extracted as described above (*n* = 2–3 biological replicates per species and condition). These RNA samples were submitted to the Keck Molecular Genomics Core for library preparation and whole-transcriptome sequencing. Quality control of RNA samples was performed using the Agilent Bioanalyzer 2100 and samples with RIN > 8 were approved for Total RNA-seq analysis. cDNA libraries were prepared using Takara SMARTer^®^ Stranded Total RNA-Seq Kit v2 (Pico Input Mammalian) and sequenced at read length of 2 × 100 cycles. Raw sequencing data was provided by Keck MGC and further analyzed using Partek^®^ Flow^®^ software, v10.0 for analysis. Within Partek^®^ Flow^®^ software, the Database for Association, Visualization, and Integrated Discovery (DAVID) software (https://david.ncifcrf.gov/) (accessed on 2 June 2022) linked to the Kyoto Encyclopedia of Genes and Genomes (KEGG) database (https://www.genome.jp/kegg/) and Ingenuity Pathway Analysis (IPA) were used for gene ontology and pathway enrichment analysis.

### 2.11. Statistical Analysis

Statistical analysis was performed using one-way ANOVA with post-hoc Tukey’s or Fisher’s LSD tests for multiple comparison or a unpaired two-tailed Student’s *t*-test for cell viability, proliferation, qPCR, ELISA, and quantification of IF staining data analysis. Total RNA-seq analysis was performed by the normalization of differentially expressed gene (DEG) counts using the DeSeq2 method within Partek^®^ Flow^®^ software. DEG counts were determined by setting an FDR or *p*-value cut-off of 0.05 and fold-changes of −2 to +2.

## 3. Results

### 3.1. Sertoli Cell Viability and Proliferation Are Dysregulated by Exposure to APAP and GEN

**Figure 1:**
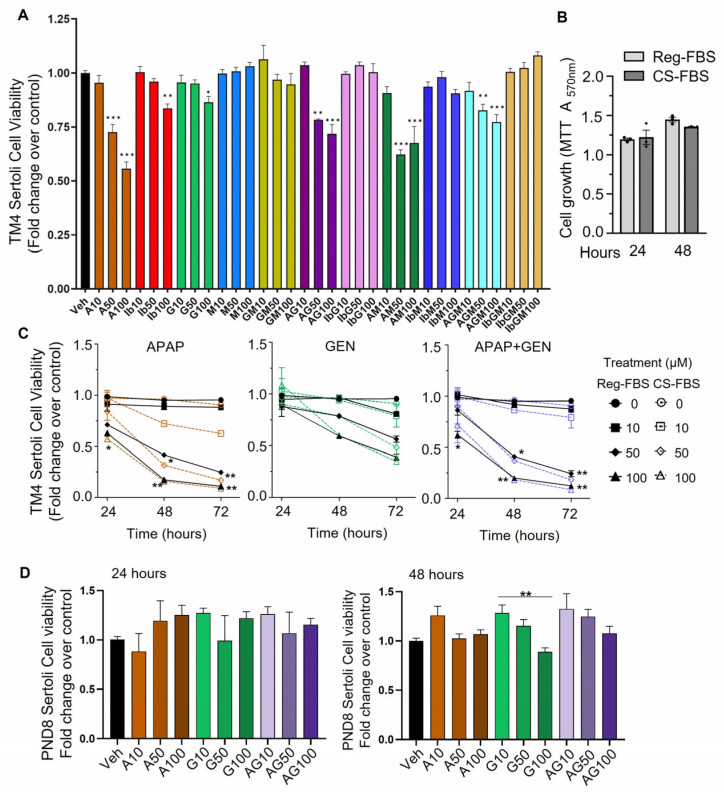
Effects of APAP, IB, GEN, and MEHP on TM4 and PND8 Sertoli cell viability measured by MTT assays. (**A**) TM4 cell viability after 24 h treatments with vehicle (Veh), APAP (A), IB, GEN (G) or MEHP (M) at 10, 50, and 100 µM, alone or mixed, in 10% Reg-FBS. (**B**) TM4 cell growth at 24 and 48 h in Reg-FBS (circles) vs. CS-FBS (diamonds) (raw absorbance values). (**C**) TM4 cell viability in medium containing 10% Reg-FBS (closed symbols) or CS-FBS (opened symbols) up to 72 h. Black: vehicle. Colors indicate treatments. (**D**) PND8 Sertoli cell viability and proliferation assessed by MTT assay after 24 h and 48 h treatments with vehicle (Veh), APAP (A), GEN (G), or their mixtures (AG) at 10, 50, and 100 µM, in 10% CS-FBS. Data are means ± SEM of three independent experiments (TM4 cells) or two experiments (PND8 Sertoli cells) performed in triplicates. A one-way ANOVA, * *p* ≤ 0.05; ** *p* ≤ 0.01; *** *p* ≤ 0.001.

Measurement of cell viability by MTT assay of TM4 Sertoli cells grown in Reg-FBS showed significant 28 to 45% decreases in viability after 24 h treatment with APAP at 50 and 100 µM, respectively. IB and GEN affected viability minimally at 100 µM, while MEHP, all IB-EDCs and GEN-MEHP mixtures had no effect on cell viability (Figure 1A). However, mixtures of APAP with EDCs at 50 and 100 µM decreased viability compared to controls but overall had less effects than APAP alone (Figure 1A). Due to how FBS contains prostaglandins (PGs) and that the effects of drugs inhibiting Cox enzymes were tested, we compared TM4 cells growth rates and responses to APAP and GEN treatments up to 72 h in regular vs. charcoal-stripped FBS, which contains less PGs than untreated FBS [13]. The growth rate of control TM4 cells over 48 h was similar in REG-FBS and CS-FBS supplemented medium (Figure 1B). Cell viability over 72 h was not affected by 10 µM APAP and GEN (Figure 1B), whereas 50 and 100 µM APAP had similar inhibitory effects on viability for both FBS types, with decreases ranging from 60 to 80% with 50 µM, and 80 to 90% with 100 µM APAP at 48 and 72 h (Figure 1C). Viability declined also with GEN, from 20 to 40% with 50 µM and 40 to 60% with 100 µM at 48 h and 72 h, similarly with both serum types (Figure 1B). APAP + GEN (AG) mixtures had effects similar to APAP alone. Considering that the type of FBS used did not noticeably impact the results, subsequent experiments were performed in a medium supplemented with CS-FBS. 

To verify that the data obtained with the mouse TM4 Sertoli cell line were applicable to primary immature Sertoli cells, we performed experiments on enriched postnatal-day (PND) 8 rat Sertoli cells, an age at which Sertoli cells are immature and non-responsive to androgen. Cell viability and proliferation were assessed at 24 h and 48 h in PND8 rat Sertoli cells treated with APAP, GEN, or APAP + GEN mixtures at 10, 50, and 100 µM, using the MTT assay. There was no significant change in any of the treatments compared to vehicle controls for both treatment times (Figure 1D). The absence of the effect of 10 and 50 µM GEN on cell viability was comparable in PND8 Sertoli cells and TM4 cells treated for 24 h, while both cell types showed non-significant decreasing trends with 100 µM GEN for 48 h. However, the two cell types responded differently to APAP, with 100 µM APAP and APAP-GEN 48 h treatments decreasing TM4 cell viability, whereas, it had no effect on PND8 Sertoli cells, suggesting that PND8 rat Sertoli cells were less sensitive to APAP exposure than TM4 cells (Figure 1C,D). Moreover, MTT assay at 48 h suggested that PND8 Sertoli cell proliferation was not affected by the treatments.

**Figure 2:**
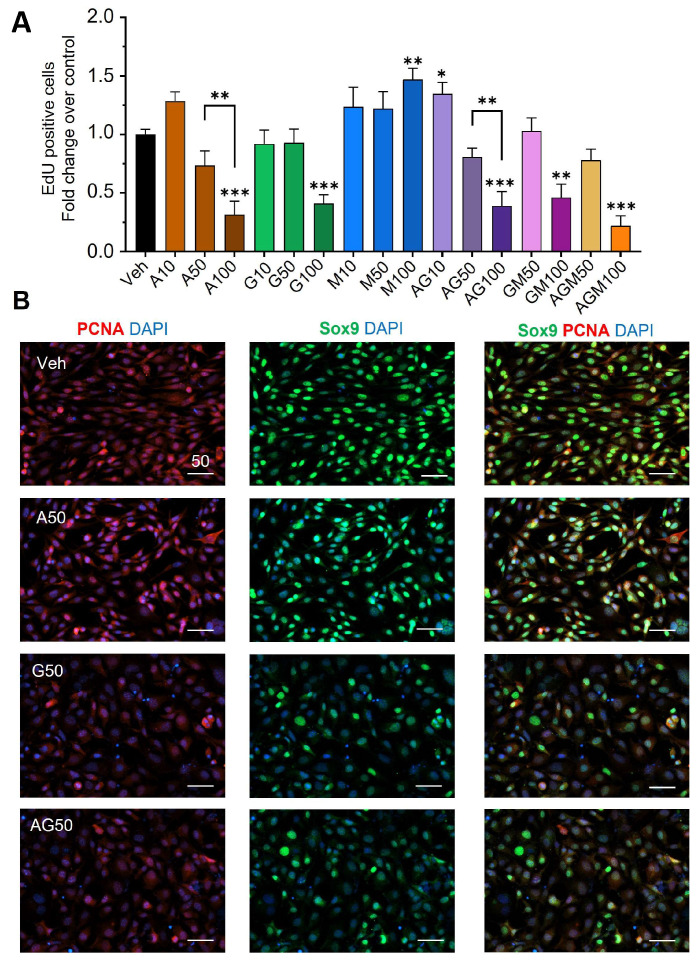
APAP and GEN but not MEHP inhibit cell proliferation and Sox9 expression in TM4 Sertoli cells. (**A**) EdU assay was performed in TM4 cells after 24 h exposure to APAP (A) and GEN (G) alone and mixed (AG) at 10, 50, and 100 µM. Data are means ± SEM of three independent experiments performed in triplicates. A one-way ANOVA, multiple comparisons; * *p* ≤ 0.05; ** *p* ≤ 0.01; *** *p* ≤ 0.001. (**B**) Representative pictures of co-IF staining of PCNA (red) and Sox9 (green) in TM4 cells treated for 24 h with 50 µM APAP and GEN, alone or in a mixture. Scale bar: 50 µm.

Cell proliferation was further measured in TM4 cells by Click-iT™ EdU assays. A 24 h treatment with APAP reduced proliferation significantly by 70% at 100 µM. GEN at ≤50 µM did not affect proliferation, whereas 100 µM reduced proliferation by 60% (Figure 2A). APAP and APAP + GEN at 10 µM slightly increased cell proliferation compared to the vehicle. At 100 µM, MEHP significantly increased proliferation by 1.5-fold but it did not modify the effects of either GEN and APAP when added to them (Figure 2A). Proliferating Cell Nuclear Antigen (PCNA) protein (red) and the Sertoli cell marker Sox9 (green) co-localized in nuclei and were decreased by 50 µM GEN and the APAP + GEN mixture, which is in agreement with the EdU data but not APAP (Figure 2B). Due to how MEHP did not decrease the proliferation and viability, the remaining experiments focused on APAP and GEN, as decreasing Sertoli cell numbers could affect germ cell development and spermatogenesis. 

**Figure 3:**
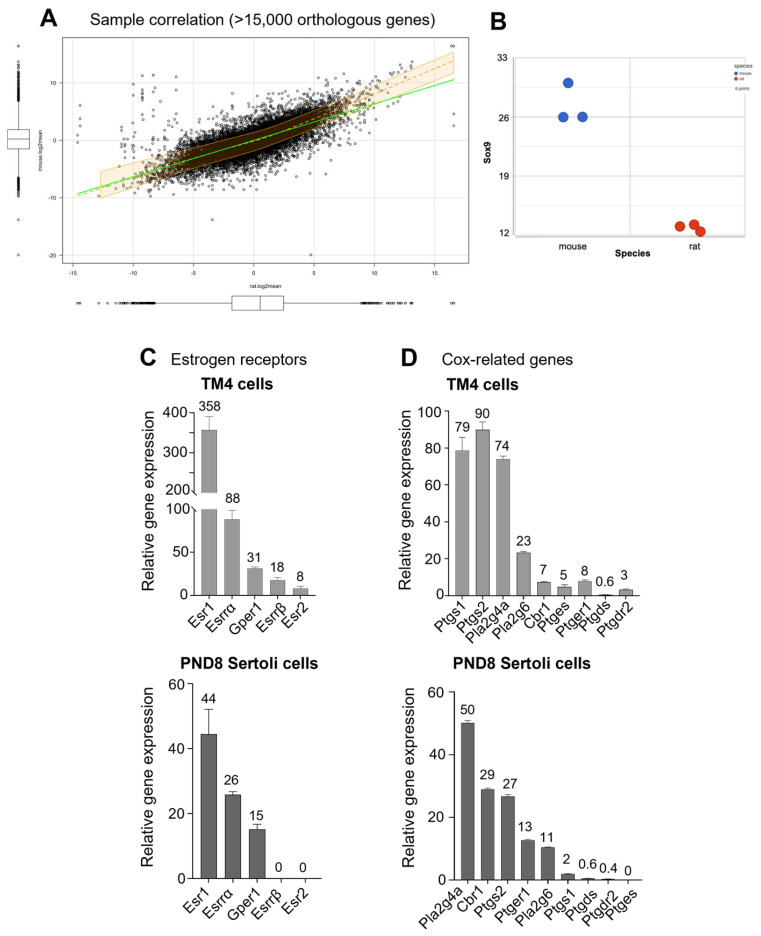
Comparison of the transcriptome and selected genes between immature mouse TM4 Sertoli cells and PND8 rat Sertoli cells. Relative gene expression was determined by RNA-seq analysis. (**A**) Correlation plot of orthologous genes between TM4 and PND8 Sertoli cells. Relative expression level of Sox9 (**B**), estrogen receptors (**C**), and Cox-related genes (**D**) in TM4 cells and PND8 rat Sertoli cells. Data are the mean ± SEM of samples from three experiments per cell type. Each cell preparation of rat Sertoli cells was obtained by pooling the Sertoli cells from 10 PND8 rat pups.

Due to how immortalized TM4 cells were generated from PND11-13 mice, a slightly more advanced age, we next compared the transcriptome of the two Sertoli cell populations by whole transcriptome sequencing (RNA-seq) of control mouse TM4 cells and PND8 rat Sertoli cells, by comparing >15,000 orthologue genes identified in mouse and rat libraries (Figure 3A). Based on the mean expression of orthologues in control samples between immature mouse and rat Sertoli cells, the linear correlation gave coefficients of 0.684 (pearson) and 0.77 (spearman). After a log2 transformation of the mean expression values, the data gave correlation coefficients for Spearman’s test of 0.774 and Pearson’s test of 0.757. These correlation coefficients indicated an overall good transcriptome similarity between the two Sertoli cell models. 

The expression levels of the Sertoli cell marker Sox9 were in relatively low abundance, between 10 and 30 RPM (Reads per million mapped reads corresponding to Sox9) in TM4 and PND8 spermatogonia (Figure 3B). A comparison across receptors binding estrogenic molecules such as GEN and selective estrogen receptor modulators showed that the ranking of relative gene abundance was similar in both cell types, although PND8 Sertoli cells expressed two to eight times less of each gene than TM4 cells. Estrogen Receptor *α* (*Esr1*, *Erα*) was the most abundant receptor in both TM4 cells and PND8 Sertoli cells, followed by the orphan Estrogen Related Receptor *α* (Essra, *Esrr-α*, *Err-α*), the G Protein-coupled Estrogen Receptor *Gper1/Gpr30*, Essrβ (*Esrrb*, *Err-β*), and the Estrogen Receptor *β* (*Esr2*, *Erβ*) (Figure 3C). 

A comparison of the relative expression of the eicosanoid pathway/Cox-related genes showed that they were expressed at similar levels in both cell types. However, *Cox1* and *Cox2* (*Ptgs1* and *Ptgs2*) were the most abundant and expressed at comparable levels in TM4 cells, whereas Pla2 and Cbr1 were the highest in PND8 Sertoli cells, and *Cox2* (*Ptgs2*) was 15 times higher than *Cox1* (*Ptgs1*) (Figure 3D). 

With the exception of the higher expression level of *Esr1* in TM4 cells, the relative gene expression levels observed in TM4 and PND8 rat Sertoli cells were within a similar range of ≤100. Overall, the comparison of the two Sertoli cell models showed a good correlation between their transcriptomes in control cells, suggesting that the two immature Sertoli cell models have comparable transcriptomes and functions, despite being from different rodent species, further validating the use of TM4 cells as surrogates for immature Sertoli cells.

**Figure 4:**
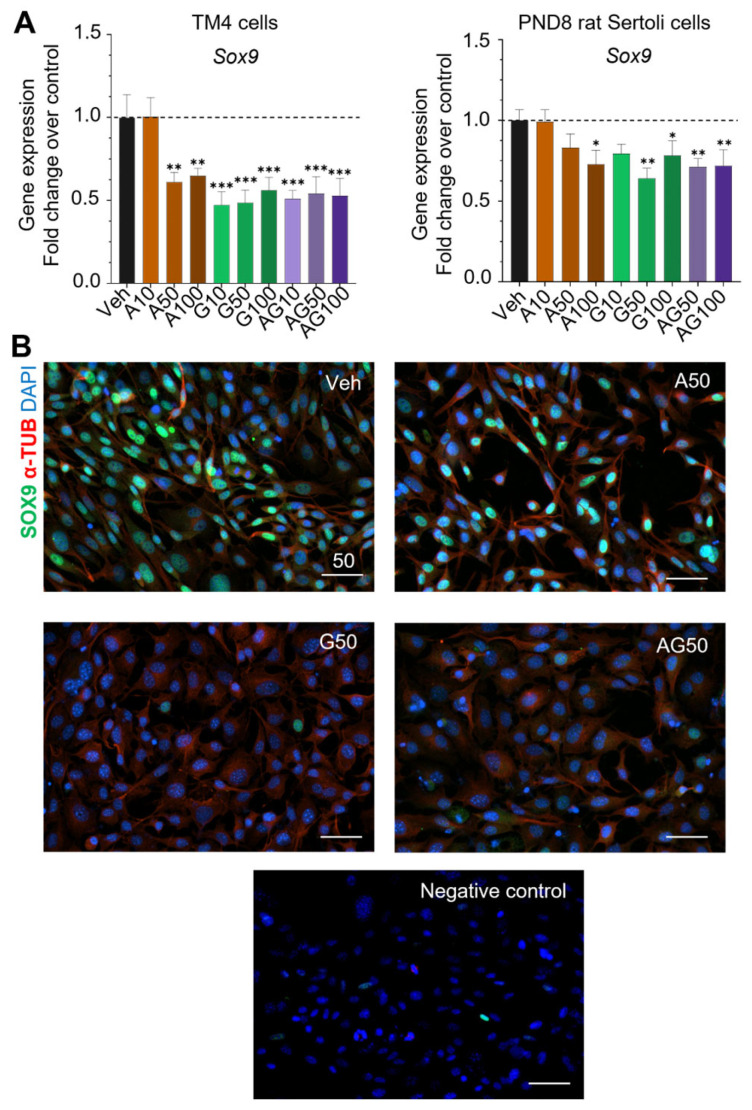
Exposure to APAP and GEN decreases Sox9 expression in mouse TM4 and PND8 rat Sertoli cells. (**A**) *Sox9* mRNA expression analyzed by qPCR after 24 h treatment with APAP (A), GEN (G), and their mixture (AG) at 10, 50, and 100 µM was observed in TM4 and PND8 rat Sertoli cells cultured in CS-FBS supplemented media. Each condition was performed in triplicate in three independent experiments and plotted as mean ± SEM. A one-way ANOVA, multiple comparisons; * *p* ≤ 0.05; ** *p* ≤ 0.01; *** *p* ≤ 0.001. Dashed line indicates the level of vehicle controls (value of 1.0). (**B**) Representative pictures of Sox9 protein IF signal (green) in TM4 cells after 24 h treatment with vehicle (Veh) or 50 µM APAP and GEN, alone or mixed. Negative control was obtained using only a secondary antibody. Cytoplasm was labelled using α-tubulin IF signal (red), contrasting with Sox9 and DAPI (blue) nuclear localization. Scale bars: 50 µm.

The expression of Sex-determination and Sertoli cell-specific marker *Sox9* was measured by qPCR analysis in cells treated for 24 h with 10 to 100 µM APAP and GEN alone or mixed, in both TM4 cells and primary PND8 rat Sertoli cells (Figure 4A). In TM4 cells, *Sox9* mRNA levels were significantly reduced by 39 and 35% of the control levels by 50 and 100 µM APAP, and by 40 to 50% in cells treated with GEN starting at 10 µM, showing toxicity at a lower dose for GEN than APAP (Figure 4A). The APAP + GEN mixture had similar effects as GEN. In PND8 Sertoli cells, 100 µM APAP and GEN at 50 and 100 µM significantly decreased *Sox9* expression by 30, 36, and 28%, respectively, and the mixture had similar effects (Figure 4A). Additionally, in TM4 cells co-stained for α-Tubulin (red) and Sox9 (green) proteins, the number of cells with Sox9 positive nuclear signal was highly reduced by 50 µM GEN and APAP + GEN mixture, in agreement with the decreases in mRNA levels (Figure 4B). The proportion of Sox9-positive cells after APAP exposure also decreased but to a lesser extent (Figure 4B). 

### 3.2. Eicosanoid Pathway Is Dysregulated in Immature Sertoli Cells Exposed to APAP, GEN, and Their Mixtures

**Figure 5:**
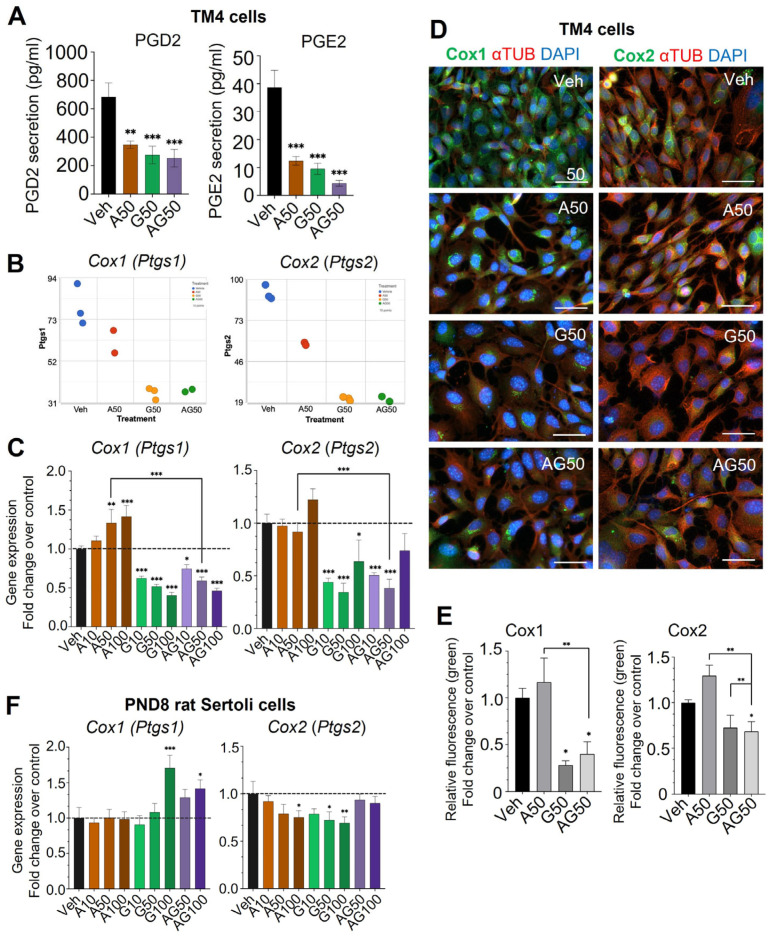
Effects of APAP and GEN on prostaglandin synthesis and Cox1 and Cox2 expression in TM4 and PND8 immature Sertoli cells. (**A**) PGD2 and PGE2 levels were measured by ELISA assays in the supernatant of cells treated for 24 h with 50 µM of APAP (A), GEN (G), and the APAP + GEN mixture (AG) solubilized in CS-FBS containing medium. (**B**) Total RNA-seq analysis showing *Ptgs1* (*Cox1*) and Ptgs2 (Cox2) in TM4 cells exposed to 50 µM APAP and GEN alone or mixed for 24 h. (**C**) mRNA expression of *Cox1* and *Cox2* measured by qPCR analysis in TM4 cells treated for 24 h with 10, 50, and 100 µM of APAP, GEN, or mixtures. Data are means ± SEM of three independent experiments performed in triplicates. A one-way ANOVA, multiple comparisons; * *p* ≤ 0.05; ** *p* ≤ 0.01; *** *p* ≤ 0.001. Dashed line indicates the level of vehicle controls (value of 1.0). (**D**) Protein expression of Cox1 and Cox2 in TM4 cells exposed to 50 µM APAP and GEN, alone or mixed, for 24 h examined by immunofluorescent staining. Green: Cox1 (left) and Cox2 (right) proteins; Red: α-Tubulin expression, used to label the cytoplasm. Blue: DAPI nuclei staining. Representative pictures are shown. Scale bars: 50 µm. (**E**) Quantification of Cox1 and Cox2 signal intensity plotted as fold-change in mean intensity signal at 488 nm compared to control. An amount of two independent experiments with duplicates per condition were performed and plotted as means ± SEM. (**F**) mRNA expression of *Cox1* and *Cox2* measured by qPCR analysis in PND8 rat Sertoli cells treated for 24 h with 10, 50, and 100 µM of APAP, GEN, or mixtures. Data are means ± SEM of three independent experiments performed in triplicates. A one-way ANOVA, multiple comparisons; * *p* ≤ 0.05; ** *p* ≤ 0.01; *** *p* ≤ 0.001. Dashed line = level of vehicle controls (value of 1.0).

APAP is known to inhibit Cox activity and decrease PG synthesis in some tissues and cells, including adult human testis [27]. Similarly, GEN was reported by us and others to alter Cox enzymes and PG synthesis in spermatogonia and prostate cancer [13,28]. Thus, we postulated that it could also be the case in Sertoli cells. Indeed, both APAP and GEN reduced the levels of PGD2 and PGE2 secreted by TM4 cells treated for 24 h with 50 µM APAP and GEN, alone and as mixtures, with GEN exerting stronger inhibitory effects. PGD2 was reduced by 49% with APAP, 60% by GEN, and 63% by the mixture, respectively (Figure 5A). APAP decreased PGE2 by 68%, GEN reduced it by 75%, and their mixture exerted the strongest inhibitory effects, with an 89% decrease of PGE2, of 4.4 pg/mL of PGE2 secreted by treated cells compared to control levels at nearly 40 pg/mL (Figure 5A). Noticeably, PGD2 concentration in control TM4 cell supernatants was 18 times higher than that of PGE2, although the transcript of Ptges was eight times higher than that of Ptgds in TM4 cells (Figure 3D), suggesting that the levels of enzymes and/or activities did not match the levels of synthase transcripts. Measurement of PGD2 and PGE2 concentrations in TM4 cells cultured in a medium with 10% REG-FBS (with higher PG contents) showed a similar inhibitory trend when exposed to 50 µM APAP or GEN alone and as mixtures (means ± SEM of PGD2 Veh: 1254 ± 379; A50: 337 ± 27; G50: 214 ± 1; AG50: 62 ± 17; means ± SEM of PGE2 values, Veh: 70 ± 18; A50: 28 ± 4; G50: 10 ± 0.2; AG50: 5 ± 0.7 for cells in Reg-FBS). 

In TM4 cells, the gene expression of *Cox1* and *Cox2* was decreased in RNA-seq analysis after 24 h exposure to 50 µM APAP and GEN, alone and as mixtures, compared to the vehicle, with stronger inhibitions induced by GEN and the mixtures (Figure 5B). Validation by qPCR analysis showed significant downregulation of *Cox1* and *Cox2* genes by GEN alone and the APAP + GEN mixture at 10 to 100 µM in TM4 cells, in agreement with the RNA-seq data (Figure 5C). However, APAP alone did not significantly decrease *Cox1* and *Cox2* expression, in contrast to RNA-seq data. This discrepancy could be due to the use of more samples for qPCR analysis and suggest more variability in the effects of APAP on TM4 cells. Protein levels of Cox 1 (green, left panels) and Cox2 (green, right panels) measured by immunofluorescence and quantified in TM4 cells showed significant decreases after exposure to GEN alone and the APAP + GEN mixture at 50 µM, in agreement with the changes in mRNA found by RNA-seq and qPCR (Figure 5D,E). APAP did not decrease but rather showed an increasing trend in Cox1 and Cox2 protein levels, in accordance with the qPCR data for APAP effects on transcripts. Notably, TM4 cell morphology was changed by GEN, with the cells and their nuclei appearing larger than in control and APAP-treated cells. 

In PND8 rat Sertoli cells, *Cox1* expression was not affected by APAP but 100 µM of GEN and the mixture caused significant upregulation of *Cox1* expression. *Cox2* expression was significantly decreased by APAP and GEN alone, while it was close to control levels with APAP + GEN mixtures at 50 and 100 µM (Figure 5F). 

Taken together, the mRNA and protein data showed that GEN disrupted more Cox1 and Cox 2 expression and PG synthesis than APAP. Moreover, the effects of GEN on Cox2 were similar in TM4 cells and immature rat Sertoli cells. 

**Figure 6:**
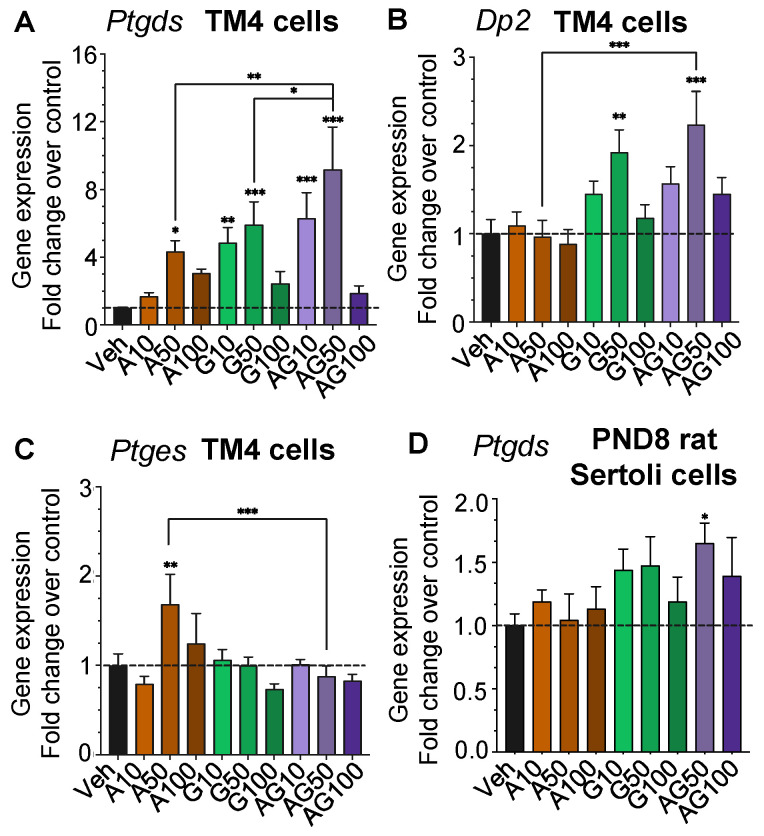
Cox-related genes are altered by exposure to APAP, GEN, and their mixtures in TM4 and PND8 rat Sertoli cells. *Ptges*, *Ptgds*, and *DP2* (*Ptgdr2*) expression was determined in cells treated for 24 h with APAP (A) and GEN (G), alone and as mixtures (AG), at 10, 50, and 100 µM. (**A**–**C**) Gene expression in TM4 cells. (**D**) Gene expression in PND8 rat Sertoli cells. Each condition was performed in triplicate in three separate experiments and presented as mean ± SEM reported. A one-way ANOVA, multiple comparisons; * *p* ≤ 0.05; ** *p* ≤ 0.01; *** *p* ≤ 0.001. Dashed lines show the level of vehicle controls (value of 1.0).

Next, we examined the expression of the PGD2 synthase, *Ptgds*, and PGE2 synthase, *Ptges*, and the receptor for PGD2, *DP2*, in TM4 cells by qPCR analysis. The expression of *Ptgds* was significantly upregulated by four- to six-fold in cells exposed to APAP at 50 µM and GEN at 10 and 50 µM and the mixtures as compared to the vehicle (Figure 6A). APAP had no effect on PGD2 receptor *Dp2* (*Ptgdr2*) but GEN and the mixture at 50 µM increased its expression by over two-fold (Figure 6B). *Ptges* expression was not significantly affected by exposure to APAP and GEN, except for a 1.8-fold increase with APAP at 50 µM (Figure 6C). 

In PND8 rat Sertoli cells, *Ptgds* expression showed upregulation in trends in cells treated with GEN alone, and it was significantly increased by the APAP + GEN mixture at 50 µM (Figure 6D). These changes were similar to the changes in *Ptgds* expression observed in TM4 cells. 

### 3.3. Estrogen Receptors Dysregulation in Immature Sertoli Cells by APAP and GEN

**Figure 7:**
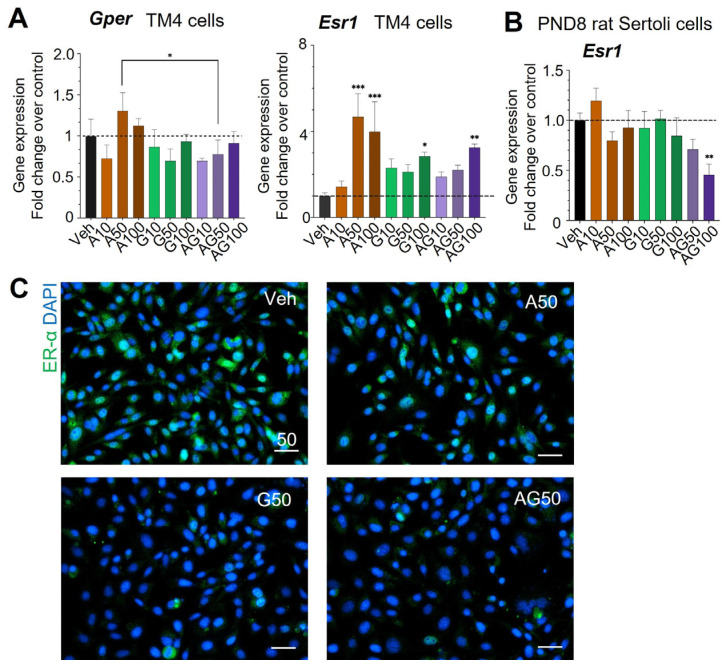
Effects of APAP and GEN on the expression of estrogen receptors in TM4 cells and PND8 rat Sertoli cells. (**A**) *Gper* and *Esr1* gene expression in TM4 cells (**B**) and *Esr1* in rat Sertoli cells after 24 h exposure to APAP and GEN alone or mixed at 10, 50, and 100 µM. Data are the means ± SEM of three experiments, each performed in triplicate for each condition. A one-way ANOVA, multiple comparisons; * *p* ≤ 0.05; ** *p* ≤ 0.01; *** *p* ≤ 0.001. Dashed lines show the level of vehicle controls (value of 1.0). (**C**) Representative pictures of immunofluorescent staining of ER-α (green) in TM4 cells treated with 50 µM of APAP, GEN, or the mixture. DAPI (blue) nuclei staining. Scale: 50 µm.

We examined next if APAP or GEN affected the expression of estrogen receptors by measuring the transcript levels of *Esr1* and *Gper* in TM4 and PND8 rat Sertoli cells by qPCR analysis. The treatments did not alter *Gper* compared to controls. The most striking effect was the four-fold increase of *Esr1* mRNA in TM4 cells treated with APAP at 50 and 100 µM, and significant 2.8-fold and 3-fold increases with 100 µM GEN and the mixture, respectively (Figure 7A). In PND8 rat Sertoli cells, *Esr1* was not affected by APAP and GEN but the mixtures induced dose-dependent decreases, with a significant 50% decrease with 100 µM APAP + GEN (Figure 7A). Immunostaining of ERα (green) in TM4 cells treated with GEN and APAP + GEN mixture at 50 µM was decreased (Figure 7C), which differed from the mRNA changes observed.

### 3.4. Dysregulation of Immature Sertoli Cell Differentiation by APAP and GEN

**Figure 8:**
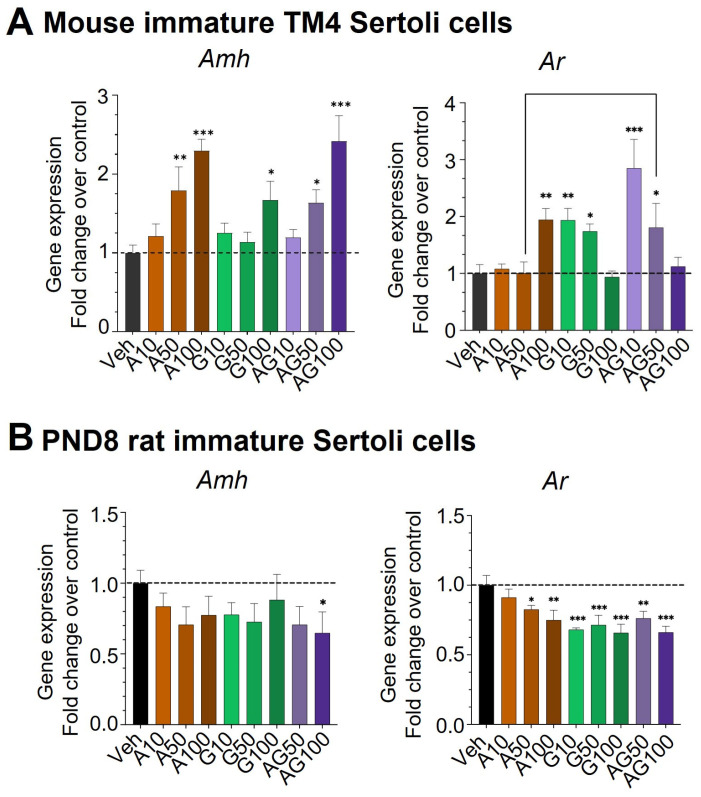
Expression of immature Sertoli cell marker *Amh* and mature Sertoli cell *Ar* in TM4 and PND8 Sertoli cells in response to APAP (A), GEN (G), and their mixtures (AG). Gene expression was analyzed by qPCR. Data are expressed as means ± SEM of three experiments with each condition performed as triplicate. A one-Way ANOVA with multiple comparisons. * *p* ≤ 0.05; ** *p* ≤ 0.01; *** *p* ≤ 0.001. Dashed lines indicate the level of vehicle control (value of 1.0).

To assess the effects of APAP and GEN on immature Sertoli cell differentiation, we measured the mRNA expression of *Amh*, a marker of fetal and neonatal immature Sertoli cells, and the Androgen Receptor *Ar*, which is expressed in differentiated mature Sertoli cells in TM4 cells and PND8 rat Sertoli cells. In TM4 cells, the gene expression *Amh* was significantly upregulated in a dose-dependent manner by APAP treatment alone and APAP + GEN mixtures at 50 and 100 µM, reaching 2.5-fold increases, and by 100 µM GEN (Figure 8A). In contrast, in PND8 Sertoli cells, *Amh* was not changed, except for a significant 40% decrease by APAP + GEN at 100 µM (Figure 8B). 

The expression of the mature Sertoli cell marker *Ar* was upregulated in TM4 cells by two-fold with 100 µM APAP and 10 and 50 µM GEN (Figure 8A), while their mixture induced an initial three-fold increase but a dose-dependent inhibition by GEN at 50 and 100 µM, respectively (Figure 8A). In PND8 rat Sertoli cells, *Ar* expression was significantly reduced by all treatments, with a maximum decrease at 40% of the control values. The difference in the responses to APAP and GEN between the two cell types suggests that the rat PND8 Sertoli cells were not at the exact same developmental stage as the mouse TM4 cells, which were generated from PND11 to 13 mice, closer to the period at which Sertoli cells initiate differentiation. This also suggests that early immature and late immature Sertoli cells may have opposite responses to APAP and GEN. 

### 3.5. APAP and GEN Disrupt TM4 Cells and PND8 Sertoli Cell Transcriptomes

**Figure 9:**
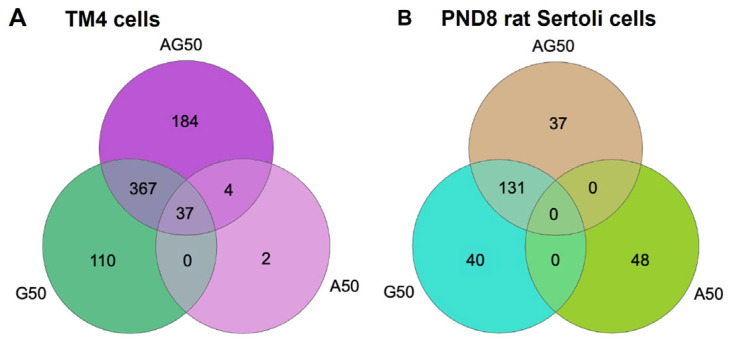
Differentially expressed genes in TM4 cells and PND8 Sertoli cells treated with 50 µM APAP (A), GEN (G), and APAP + GEN (AG) mixture. RNA-seq analysis was used to analyze the transcriptomes of immature TM4 cells and PND8 rat Sertoli cells treated with APAP and GEN alone or mixed. Venn diagrams display statistically significant differentially expressed gene (DEG) counts using false discovery rate or *p*-value ≤ 0.05 with a cutoff range of −2 to +2 in Partek Flow.

To identify differentially expressed genes (DEGs) and key molecular mechanisms and functional pathways dysregulated by APAP and GEN, we analyzed the transcriptomes of TM4 cells and PND8 Sertoli cells by Total RNA-seq analysis, followed by pathway analysis with KEGG and Ingenuity Pathway Analysis (IPA), in cells treated for 24 h with a vehicle or 50 µM APAP (A), GEN (G), or their mixture (AG). Venn diagrams representing DEG count for each treatment type in each Sertoli cell model, and 704 DEGs were found in TM4 cells and 256 DEGs were found in PND8 Sertoli cells for all treatments together, with most DEGs occurring in cells treated with GEN and the APAP + GEN mixture (Figure 9A,B; full list in Supplementary Table S1). These data indicated a heightened sensitivity of immortalized TM4 cells to GEN compared to PND8 Sertoli cells (110 GERs in TM4 cells vs. 40 DEGs in PND8 Sertoli cells, respectively) (Figure 9A,B). The results also implied that PND8 Sertoli cells had similar susceptibility to APAP and GEN (48 vs. 40 DEGs, respectively). DEGs comparison suggested that the effects of mixtures were mainly driven by GEN in both cell types. Indeed, in TM4 cells, there were only two DEGs unique to APAP treatment, four in common between the APAP and AG mixture, and thirty-seven common to the APAP, GEN, and AG mixture. The majority of DEGs were attributed to GEN and the AG mixture (Figure 9A). In PND8 Sertoli cells, APAP exerted a distinctive effect with 48 unique DEGs, and none shared with GEN or the mixture (Figure 9B). Most DEGs in PND8 cells were found with the GEN and AG mixture, with 63% being common to both. This hinted at the absence of interaction between the eicosanoid and estrogen pathways in PND8 Sertoli cells, whereas the two pathways shared common targets in TM4 cells. Overall, these data supported the qPCR results on specific Sertoli cell-related genes.

The 10 most up- and down-regulated genes by 50 µM APAP, GEN, or their mixture in both cell types showed a higher amplitude of fold-changes with GEN and the mixture in TM4 cells than in PND8 Sertoli cells. Despite sharing common target pathways, the most up- and down-regulated DEGs were different between cell types (Table 2). Noticeably, *Ereg*, a growth factor of the EGF family secreted by Sertoli cells [29], was decreased by five-fold with APAP but by 15- and 18-fold by the GEN and AG mixture, respectively. Similarly, *Hbegf* (Heparin-Binding EGF-Like Growth Factor), which is involved in the ErbB signaling pathway and Akt regulation, was downregulated by three-fold with APAP but twelve-fold by GEN and the mixture. Since AKT is involved in the regulation of immature Sertoli cell proliferation and anti-apoptosis [30], decreases in proteins regulating its activity could antagonize immature Sertoli cell functions. Other DEGs were unique for one treatment, such as *Lif* (Leukemia Inhibitory Factor), secreted by Sertoli cells under TNFa control and important for SSC survival [2] that decreased by three-fold due to APAP only. Another important factor produced by Sertoli cells is the chemokine *Ccl20* (CC-chemokine ligand 20) which was reduced by 10- and 13-fold by GEN and the mixture, respectively. Since Ccl20 was recently shown to be released in Sertoli cell exosomes that regulate Leydig cell survival [31], reducing its production by Sertoli cells could hinder Leydig cell function. Another gene downregulated by ~nine-fold in GEN-treated TM4 cells is *Usp18* (Ubiquitin specific protease 18), shown to promote proliferation and be negatively regulated by Wt1 (Wilms tumor gene), a transcription factor that plays a role in spermatogenesis [32,33] and in inhibiting interferon signaling [34]. Thus, the Ups18 reduction could have multiple consequences. The most decreased gene was *Fosl1*, part of the transcription factor AP-1 complex that was reduced by 15- and 22-fold by the GEN and mixture, respectively. Fosl1 is normally upregulated in immature Sertoli cells [35], thus, its decrease could perturb their development. Among upregulated genes, the gene *Greb1* (growth regulating estrogen receptor binding 1), which is regulated by Esr1 in TM4 cells and primary Sertoli cells [36] and is involved in hormone-responsive breast and prostate cancers, was increased by 3.5-fold only by the APAP + GEN mixture. However, its function has yet to be discovered. More is known about the role of the cholesterol esterifying enzyme Acat2 (Acetyl-CoA Acetyltransferase 2) which was increased by three-fold due to APAP and five-fold by the GEN and mixture. Acat2 is known to play a role in cholesterol homeostasis in testis [37] and its increase could dysregulate cholesterol in Sertoli cells. 

In PND8 Sertoli cells, the most decreased gene by APAP (by 13-fold) was Hoxb1 (Homeobox b1), a transcription factor involved in morphogenesis and associated with gene repression [38]. The transcription repressor E2F8 was decreased by GEN and the mixture by more than five-fold. This gene is involved in some cancers and in the switch from mitosis to meiosis in female germ cells [39]. However, its role in Sertoli cell is unknown. Cdca3 (Cell division cycle associated 3) was also decreased by ~five-fold by GEN and the mixture. Increase in this gene is associated with poor prognosis in several cancers but nothing is known for Sertoli cells. The most upregulated gene in PND8 Sertoli cells is Gdf15 (growth differentiation factor 15), a member of the TGFb family that is secreted and activates the Smad signaling cascade. It has been shown to be involved in cell repair, to be increased in inflammation and oxidative stress, and in testicular cancer [40]. A gene known for its critical role in connecting Sertoli and germ cells and regulating Sertoli cell development and spermatogenesis is the Gap junction alpha-1 protein (Gja1; Connexin 43) [41]. In this study, Gja1 KD in mice was shown to delay Sertoli cell maturation and aberrantly maintain their proliferation in adulthood. In the present study, Gja1 was upregulated by ~four-fold in GEN-treated TM4 cells, suggesting that it could participate to early Sertoli cell maturation, as suggested by the finding of androgen receptor increase in GEN-treated cells compared to control cells (Figure 8A). Interleukin 6 (Il6) is another gene increased by 5-fold and 2.7-fold by GEN and the mixture, respectively. Ptx3 (pentraxin 3), which increased by 2.6-fold with GEN, is also involved in inflammation and cancer [42]. It is expressed in the male reproductive tract and semen [43]. 

KEGG analysis for pathway enrichment and functions commonly altered in TM4 Sertoli cells and PND8 rat Sertoli cells exposed to 50 µM APAP + GEN mixture highlighted terms including viral carcinogenesis, necroptosis, transcriptional misregulation in cancer, the p53 signaling pathway, cellular senescence, the TNF-signaling pathway, and pathway involving protein interaction with cytokine and a cytokine receptor (Table 3).

It is noticeable that the shared pathways suggest that the mixture of APAP and GEN (mainly driven by GEN) disrupts genes related to cancer and inflammation. Pathways exclusive to TM4 cells exposed to AG mixture included Ribose biogenesis, AMPK, NF-kappa β, IL-17, Steroid biosynthesis, and TGF-β signaling, some known to play a role in Sertoli cell functions (Table 4). Pathways targeted by 50 µM APAP + GEN mixture in PND8 rat Sertoli cells comprised Foxo, PI3K-Akt, and JAK-STAT signaling pathways, and were also involved in Sertoli cell functions (Table 5). IPA database highlighted downregulated signaling molecules and predicted an inhibition in the estrogen receptor and eicosanoid related pathways in TM4 cells treated for 24 h with 50 µM APAP + GEN mixture (Supplementary Figure S2A,B). Some of the genes that were the most up- or down-regulated fitted within these functional categories, with genes decreased by GEN potentially related to disrupted Sertoli cell functions, whereas some of the upregulated genes suggested inflammatory processes.

## 4. Discussion

### 4.1. Dysregulation of Sertoli Cell Development by APAP and GEN

The goal of this study was to examine whether exposing infants to common EDCs and frequently used antipyretic/analgesic drugs could present a risk to the developing male reproductive system by altering immature Sertoli cell functions and whether concomitant exposures would have different outcomes than individual compounds. Although the first set of experiments and the analysis of gene markers of Sertoli cells included APAP, IB, GEN, and MEHP as treatments, in view of the minimal or no effects observed with ibuprofen and MEHP, we decided to focus on APAP and GEN, which both exerted significant effects on TM4 Sertoli cells. The present study showed that exposure to APAP at a concentration in the range of levels measured in children [23] decreased the viability and dysregulated cellular proliferation of immature TM4 Sertoli cells, while it altered the expression of genes important for Sertoli cell functions both in TM4 cells and PND8 Sertoli cells. While the cytotoxic effect of APAP alone and in mixtures was clear at 50 µM, similar to levels measured in childrens’ blood upon treatments with recommended doses, a concentration of 100 µM killed most cells by 48 h treatment. In contrast, GEN exerted mainly cytostatic effects, as shown by minimal effects on the viability concomitant to decreases in proliferation. Such cytostatic effects of GEN were reported for other testicular cells, including our study on the C18-4 undifferentiated spermatogonial cell line [13]. While several studies have shown that in utero or perinatal exposure to APAP dysregulates male reproductive development [14,44] and exerts intergenerational effects on testes [18], there are no current studies comparing the effects of EDCs in combination with exposure to analgesic drugs in infants [3]. The present study highlights effects induced by either APAP or GEN individually, identifying functions, genes, and functional pathways more susceptible to either compound and also identifying genes that are more disrupted by the combination of the two compounds, further contributing to the current knowledge on possible origins of male infertility.

The finding that the immature Sertoli cell marker *Amh* was upregulated by APAP, GEN, and the mixtures in TM4 cells in a dose-response manner suggested the dysregulation of the developmental program of the cells. In this case, APAP acted at a lower dose than GEN, suggesting a role for the eicosanoid pathway in Amh expression whereas the effects of GEN may have been due to intracellular signaling in response to high estrogen exposure [45]. The fact that the mixture effects were similar to those of each individual compound suggests that both APAP and GEN disrupted Amh production via the same mechanism. The upregulation by APAP alone suggests that APAP exposure contributes to increased immature Sertoli cell signature [46]. Interestingly, *Amh* dysregulation has been associated with reproductive disorders, including Sertoli cell-only tubules, Leydig cell hyperplasia, and Mullerian duct syndrome [46,47,48].

While the expression of mature Sertoli cell marker *Ar* in TM4 cells was increased only by the highest dose of APAP, it increased starting at 10 µM with GEN and the mixtures, indicating a high sensitivity of the cells to GEN, a soy phytoestrogen to which many babies are exposed via soy-based formula. Our data reveals that a low dose of 10 µM GEN, similar to the blood levels found in soy-formula-fed babies [24] was sufficient to disrupt the expression of genes important for Sertoli cell functions such as Sox9 and several eicosanoid pathway genes, including Cox2 in TM4 and PND8 Sertoli cells, which might be a concern. Our observations that APAP alone had no effect at a low dose but that the largest increase in Ar was found with the mixture suggests a synergistic trend between GEN and APAP on Ar expression at a low dose, with a dose-dependently decreased to basal levels only seen with the mixtures. Together with the decreases in cell proliferation, the negative correlation between *Amh* and *Ar* expression observed with the mixtures could reflect a disruption of the proliferation needed to establish adequate numbers of Sertoli cells, as well as alteration of Sertoli cell maturation process, causing functional impairment that could have deleterious consequences later in life [46,49].

### 4.2. Similarities and Differences between TM4 Cells and PND8 Immature Sertoli Cells

The upregulation of Amh and Ar in TM4 cells treated with APAP and GEN contrasted with the downregulation of both genes in PND8 Sertoli cells, suggesting differential effects, despite a close similarity in the trascriptomes of the two Sertoli cell models, and their comparable responses for Sox9 gene. As a hallmark Sertoli cell marker, *Sox9* gene expression in TM4 cells and PND8 rat Sertoli cells were similarly decreased, suggesting that the TM4 cell line recapitulates some of the effects that APAP and GEN might exert on primary non-immortalized immature Sertoli cells. Sox9 protein expression also decreased in TM4 cells exposed to GEN and the mixture, validating the results observed on transcript levels. Sox9 is a transcription factor important in sex determination and involved in Sertoli cell maturation and expressed in adult testis via Sertoli cells [50,51]. Some of the differences observed between the two Sertoli cell models could be due to the fact that TM4 cells were generated from late juvenile mice (PND11-13) [52], when some Sertoli cells start entering the maturation process, compared to the immature primary Sertoli cells isolated from PND8 rats, an age at which rat Sertoli cells are all immature. It could also reflect the fact that TM4 are immortalized cells, a process that can alter developmental processes, compared to primary cells undergoing active and dynamic developmental changes. Although it is generally accepted that mice do not express androgen binding protein (ABP) which binds testosterone in the testis, one study in CD1 mice testes reported that the ABP gene was expressed but at a much lower extent than what is observed in a rat, leading to significantly lower levels of ABP protein produced in mice [53]. The discrepancy between Ar expression in TM4 cells and primary rat Sertoli cells could be related to their difference in ABP levels, with mouse testis containing higher levels of free testosterone available for its receptor and possible feedback effects.

A noticeable difference between TM4 and PND8 Sertoli cells is the levels of estrogen receptors in basal conditions and in response to APAP and GEN. Total RNA-seq analysis of TM4 confirmed the lack or very low level of *Esr2* expression in both cell types. This is also supported by studies that suggested that ER-α and ER-β expression in rat Sertoli cells changed depending on age [54]. Other studies reported the effects of the non-steroidal mycotoxin Zearalenone on TM4 cells, described as estrogen-responsive via *Esr1* and *Gper1* expression [55]. Zearalenone was suggested to act as xenoestrogen mediating Sertoli cell differentiation through increases in ROS production via the MAPK pathway. The extent of dysregulation to TM4 cells and primary PND8 rat Sertoli cells following exposure to the common analgesic/antipyretic APAP, and the frequent dietary exposure to GEN suggest the need to address how dysregulation occurs when concomitant exposures to APAP and GEN take place, further complicating the assessment of possible reproductive harm at fetal or perinatal ages [56].

### 4.3. Is There a Link between APAP and GEN Effects on Sox9 and Eicosanoid Pathway?

The decreased levels of Sox9 in TM4 cells as well as in PND8 immature rat Sertoli cells, together with other shared endpoints, infer that APAP and GEN might alter immature Sertoli cell function and possibly induce a delay in Sertoli cell maturation. This finding coincides with Rossitto and colleagues, who observed the F0 offspring of pregnant mothers who received APAP alone had a slight decrease in Sox9 expression compared to the control [18]. Moreover, Sox9 is suggested to play a major role in L-PGDS/PGD2 pathway during fetal development [57,58,59,60]. In fetal mice at 13.5 days post-coitum (dpc), the ablation of Sox9 caused decreased production of L-PGDS and, therefore, the accumulation of PGD2 in mouse testis was reduced [57]. Our findings of decreased *Sox9* expression in both TM4 cells and PND8 Sertoli cells after exposure to APAP and GEN alone or as mixtures, and the concomitant decreases of PGD2 and PGE2, suggest that both processes could also be related in our models. Moreover, the increased expression of *Ptdgs* in TM4 cells could be a compensatory response to the reduced levels of PGs and Coxs genes and proteins. PGD2 was found to be crucial during development and reproduction [61] as it interacts with L-Pgds and Sox9, facilitating normal Sertoli–germ cell interaction and acting independently of fibroblast growth factor 9 (FGF9)/Sox9 regulatory loop. One can speculate that the upregulation of PGD2 receptor DP2 in TM4 cells by GEN and mixtures corresponds to a compasatory upregulation of the whole PGD2 pathway in response to the decreases in PGs.

Changes in the expression of *Cox2* and *Ptgds* in response to GEN in PND8 rat Sertoli cells suggested some synonymous trends with TM4 cells. Both Cox genes showed mostly decreased expression by exposure to GEN alone and mixed by RNA-seq analysis, qPCR, and protein detection in cells. However, there were some discrepancies for APAP alone between the RNA-seq and qPCR data, which may be due to the different samples used in these experiments. The downregulation trends were more prominent in TM4 cells but there were nonetheless similar decreases for *Cox2* and increases for *Ptgds* between TM4 cells and PND8 rat Sertoli cells. Kristensen et al. studied the effects of exposure to EDCs and analgesic drugs such as the xenoestrogen bisphenol A (BPA) and APAP on the synthesis of PGD2 in the juvenile Sertoli cell line SC5 [62]. In that cell model, PGD2 levels were shown to be decreased by APAP or BPA alone, similar to our findings with APAP and the phytoestrogen GEN in TM4 cells.

### 4.4. Mechanisms Dysregulated in Immortalized and Primary Immature Sertoli Cells

Major functional pathways altered in both TM4 and PND8 rat Sertoli cells identified by KEGG pathway enrichment analysis included p53, and TNF signaling pathways, based on treatments with 50 µM APAP + GEN mixture. Genes highlighted in alcoholism were related to ethanol signaling to histones H3 and H4 acetylation that involve genes such as Hdac5, H2ac12, H3c7, and H4c3. Histone deacetylases mediate reactive oxygen species (ROS) production which is also controlled by PGD2 as an adaptive stress mechanism to promote cell survival in TM4 cells [63]. p53 signaling was detected in both Sertoli cell types which included the downregulation of *Cdkn1a* and *Ccnd1* by exposure to AG50 whereas *Cdkn1a* and *Mdm2*, which mediate oncogene activation, were upregulated in PND8 rat Sertoli cells. Elucidating these signaling discrepancies between TM4 and PND8 rat Sertoli cells will help in further identifying new target genes and pathways possibly involved in cases of male infertility.

Using the KEGG database for pathway analysis in TM4 cells identified AMPK, NF-kappa β, and TGF-β signaling as targets. While steroid biosynthesis was affected in TM4 cells, one can speculate that it may be due to the dysregulation by both GEN and APAP. Prolonged exposure to APAP in a xenograft model of human fetal testis was reported to decrease testosterone production [64], likely due to alterations of steroidogenic enzymes *Cyp11a1* and *Cyp17a1* expression. In our total RNA-seq analysis performed on postnatal models corresponding to juvenile ages, DEG counts showed that the 24-dehydrocholesterol reductase gene (*Dhcr24*) was upregulated after exposure to GEN and the mixture, which is key in cholesterol biosynthesis (Supplementary Table S1). The cholesterol esterifying enzyme Acat2 was also upregulated (Table 2). Considering that cholesterol is the key precursor to steroidogenesis [6,65,66] and that Sertoli cells produce estrogens from androgens synthesized by Leydig-cells after birth (as well as before birth in human and rat), the unregulation of enzymes involved in steroid production may lead to disrupted Sertoli cell functions, including the regulation of germ cells.

Most DEG counts identified by RNA-seq analysis in TM4 cells were due to G50 and AG50 treatments in TM4 and PND8 Sertoli cells. The molecular mechanisms highlighted in PND8 rat Sertoli cells exposed to AG50 included PI3K-Akt, and JAK-STAT signaling, which are important in Sertoli cell proliferation [67,68]. Moreover, Sertoli cell proliferation was proposed to be mediated via NF-kappa Β signaling in a PI3K-Akt and ERK1/2-dependent manner, stimulated by the binding of estradiol on ER-α in 15-day-old rats [54]. The altered pathways identified in the current study corroborate the possible involvement of NF-kappa β signaling in Sertoli cell proliferation, highlighted in TM4 cells, and PI3k-Akt and ERK1/2 signaling in PND8 rat Sertoli cells. GEN action was also shown to be mediated in fetal mouse testes by interaction with ER-α, supported by diminished inhibitory effects of GEN in ER-α knockout mice [69]. Here, we found that GEN upregulated *Esr1* expression in TM4 cells but not in PND8 Sertoli cells. The differences in Esr1/ER-α expression in our models could be attributed to age differences between postnatal ages [54], as well as with fetal ages. In comparison, we showed that exposure to GEN alone and the mixtures caused the downregulation of GPER expression in TM4 cells. A comparative study on GPER, ER-α, and ER-β expression in Sertoli cells derived from 5-, 15, and 120-day-old rats found that while ER-α/ER-β played a role in Sertoli cell proliferation, *Gper* expression mediated by E2 binding was involved in apoptotic signaling [70,71]. Future studies of *Gper* expression along with apoptotic markers such as Bax and Bcl2 after exposure to GEN and APAP + GEN mixtures in TM4 cells may elucidate the possibility that GPER-mediated estrogen signaling could balance cell proliferation and apoptosis processes. The comparison of the data obtained between TM4 and PND8 Sertoli cells should further be nuanced by the knowledge that the functional pathways identified here have been shown in other models such as cancers to crosstalk with each other. This is the case with NF-kappa Β and Stat3, found to crosstalk and promote the progression of several cancer types [72].

## 5. Conclusions

This study attempted to bridge the gap of knowledge that exists on the possibility that effects of EDC and analgesic drug exposure on male infants may contribute to male reproductive disorders such as infertility by disrupting Sertoli cells, essential to spermatogenesis. Using two models of immature rodent Sertoli cells, our results showed that Sertoli cell function and development were dysregulated by exposure to APAP and GEN alone and as mixtures in both TM4 cells and PND8 rat Sertoli cells in culture. Gene expression studies overall highlighted similar effects by APAP and GEN on critical genes and biological functions and on Cox-related genes. These data highlight the need for caution while exposing infants to analgesic drugs such as APAP, and the possibility that exposure to estrogenic EDCs such as GEN might also exert adverse effects. This is further emphasized by the fact that the concentration of 50 µM of APAP and GEN used in this study were in the range of those measured in the blood of human babies, who may be exposed more frequently to APAP for pain or fever treatment, and GEN from a soy-containing diet, than the 24 h time exposure used here. While animal studies do not always extrapolate to humans, there are epidemiological studies supporting the concern that these chemicals can adversely affect humans. Furthermore, the likelihood that such chemicals acting on different molecular pathways might have common functional and gene targets should be considered while evaluating the potential reproductive toxicity of these compounds. Additionally, p53, TNF, and TGF-β signaling pathways detected as targets in both Sertoli cell models herein provide a snapshot of possible mechanisms in Sertoli cells that may be involved in male infertility.

## Figures and Tables

**Table 1:** Primer sets for q-PCR analysis.

Gene	Forward Primer	Reverse Primer	Product Size (bp)
Rat			
*Gapdh*	CCATTCTTCCACCTTTGATGCT	TGTTGCTGTAGCCATATTCATTGT	98
*Sox9*	TGCTGAACGAGAGCGAGAAG	ATGTGAGTCTGTTCGGTGGC	160
*Amh*	GTGGGTGGCAGCAGCACTAGG	CGGGCTGTTTGGCTCTGATTCCCG	69
*Ar*	CGGTCGAGTTGACATTAGTGAAGGACC	ATTCCTGGATGGGACTGATGGT	66
*Cox1*	AGGTGTACCCACCTTCCGTA	GGTTTCCCCTATAAGGATGAGGC	242
*Cox2*	ACGTGTTGACGTCCAGATCA	CTTGGGGATCCGGGATGAAC	234
*Ptgds*	ATTCAAGCTGGTTCCGGGAG	CAGGAACGCGTACTCATCGT	242
*Esr1*	GCCACTCGATCATTCGAGCA	CCTGCTGGTTCAAAAGCGTC	107
*Ppar-α*	TGCGGACTACCAGTACTTAGGG	GCTGGAGAGAGGGTGTCTGT	72
Mouse			
*Rps29*	TGAAGGCAAGATGGGTCAC	GCACATGTTCAGCCCGTATT	127
*Cox1*	CCTCTTTCCAGGAGCTCACA	TCGATGTCACCGTACAGCTC	70
*Cox2*	CAGGACTCTGCTCACGAAGG	ATCCAGTCCGGGTACAGTCA	231
*Ptgds*	GGCTCCTGGACACTACACCT	CTGGGTTCTGCTGTAGAGGGT	160
*Ptges*	GGCTCCTCCAAAGACGGAAA	TGGCACAGCATGGGTCTTAG	226
*Ptgdr2*	CACGTGTCGGTGCTGTTG	GATGAGTCCGTTTTCCACCA	63
*Amh*	GGGGAGACTGGAGAACAGC	AGAGCTCGGGCTCCCATA	67
*Ar*	ACCAGATGGCGGTCATTCAG	TGTGCATGCGGTACTCATTG	135
*Sox9*	TCGGACACGGAGAACACC	GCACACGGGGAACTTATCTT	96
*Esr1*	TCTCCTCAAACACATCCCGTG	GGCGAGTTACAGACTGGCTC	96
*Gper*	CCTGGACGAGCAGTATTACGATATC	TGCTGTACATGTTGATCTG	77
*Err-α*	CGCTGTCAGCTGGAGGAA	ACCTTGGGCTCCGGCA	199
*Err-β*	TCTTCCCAGCTCCCACAGTA	CCCCATGCAAGCTTCGTAGT	106

**Table 2:** List of Down- and Up-Regulated Genes in TM4 and PND8 Sertoli Cells Treated with 50 µM APAP, GEN, or their Mixture for 24 h Compared to Controls, Identified by RNA-seq Analysis.

TM4 APAP (50 µM)					
Downregulated			Upregulated		
Gene	*p*-value	Fold change	Gene	*p*-value	Fold change
*Ereg*	2.36 × 10^−11^	−4.90	*Dag1*	1.13 × 10^−4^	2.09
*Hbegf*	8.14 × 10^−10^	−3.37	*Rpa1*	1.29 × 10^−4^	2.11
*Lif*	1.39 × 10^−7^	−2.96	*Ubc*	2.08 × 10^−7^	2.20
*Cth*	1.14 × 10^−11^	−2.84	*Mki67*	1.08 × 10^−14^	2.29
*H1f1*	4.41 × 10^−18^	−2.55	*Kif20a*	4.78 × 10^−6^	2.32
*H1f0*	3.34 × 10^−21^	−2.54	*Ckap2l*	2.18 × 10^−6^	2.43
*Il1rl1*	5.63 × 10^−8^	−2.51	*Acat2*	1.76 × 10^−4^	3.28
*Mtmr10*	4.93 × 10^−5^	−2.51	*Mettl26*	1.93 × 10^−5^	8.46
*H4f16*	4.61 × 10^−10^	−2.39			
*Prkg2*	2.33 × 10^−10^	−2.35			
**PND8 rat Sertoli APAP (50 µM)**					
**Downregulated**			**Upregulated**		
Gene	*p*-value	Fold change	Gene	*p*-value	Fold change
*Hoxb1*	4.11 × 10^−2^	−13.14	*Ppp1r3d*	3.85 × 10^−2^	12.17
*Senp18*	3.59 × 10^−2^	−11.41			
*Hjv*	3.63 × 10^−2^	−10.06			
*Sypl2*	4.26 × 10^−2^	−7.85			
*Hs3st2*	3.37 × 10^−2^	−7.70			
*Mal*	4.63 × 10^−2^	−7.57			
*Nmur2*	4.35 × 10^−2^	−7.48			
*Esrrg*	4.85 × 10^−2^	−5.88			
*Synb*	4.50 × 10^−2^	−5.66			
*Akain1*	4.52 × 10^−2^	−5.23			
**TM4 GEN (50 µM)**					
**Downregulated**			**Upregulated**		
Gene	*p*-value	Fold change	Gene	*p*-value	Fold change
*Fosl1*	5.86 × 10^−28^	−15.38	*Akr1c14*	1.29 × 10^−18^	3.57
*Ereg*	6.71 × 10^−35^	−14.81	*Mboat2*	8.25 × 10^−8^	3.85
*Tgtp1*	1.06 × 10^−17^	−13.57	*Gja1*	3.97 × 10^−38^	3.89
*Gbp3*	8.73 × 10^−60^	−13.20	*Dhcr24*	4.89 × 10^−5^	4.01
*Tgtp2*	1.37 × 10^−19^	−13.16	*Nbl1*	1.07 × 10^−5^	4.17
*Hbegf*	1.64 × 10^−42^	−12.08	*Scd2*	1.10 × 10^−72^	4.88
*Ccl20*	3.98 × 10^−5^	−10.50	*Lars2*	9.28 × 10^−5^	5.29
*Ifit3*	6.09 × 10^−85^	−9.99	*Acat2*	3.36 × 10^−9^	5.35
*Iigp1*	2.80 × 10^−36^	−8.57	*Mettl26*	3.78 × 10^−6^	7.98
*Usp18*	1.67 × 10^−54^	−8.45	*Mettl7a1*	2.43 × 10^−11^	8.48
**PND8 rat Sertoli GEN (50 µM)**					
**Downregulated**			**Upregulated**		
Gene	*p*-value	Fold change	Gene	*p*-value	Fold change
*Depdc1b*	7.54 × 10^−4^	−10.00	*Ptx3*	1.17 × 10^−43^	2.62
*H2ac10*	1.39 × 10^−37^	−7.87	*Mdm2*	5.71 × 10^−67^	2.93
*E2f8*	4.22 × 10^−8^	−5.69	*Bbc3*	2.14 × 10^−21^	3.02
*Sgo2*	2.73 × 10^−6^	−4.99	*Csf2rb*	6.13 × 10^−5^	3.17
*Cenpq*	1.56 × 10^−3^	−4.96	*Cd80*	4.64 × 10^−9^	3.36
*Pbk*	1.09 × 10^−4^	−4.94	*Abcb1b*	2.99 × 10^−101^	3.66
*Cdca3*	8.23 × 10^−8^	−4.71	*Star*	2.53 × 10^−188^	4.36
*Depdc1*	1.17 × 10^−4^	−4.71	*Cdkn1a*	1.35 × 10^−155^	4.86
*Hist1h2bc*	7.92 × 10^−5^	−4.67	*Il6*	3.29 × 10^−8^	5.18
*Uhrf1*	1.10 × 10^−21^	−4.44	*Gdf15*	2.02 × 10^−22^	9.17
**TM4 APAP + GEN (50 µM)**					
**Downregulated**			**Upregulated**		
Gene	*p*-value	Fold change	Gene	*p*-value	Fold change
*Fosl1*	1.19 × 10^−27^	−22.36	*Greb1*	4.85 × 10^−12^	3.54
*Ereg*	2.19 × 10^−31^	−17.81	*Thra*	3.82 × 10^−6^	3.60
*Mx1*	3.19 × 10^−8^	−15.73	*Nbl1*	3.52 × 10^−4^	3.63
*Il1rl1*	1.39 × 10^−53^	−15.63	*Ulk1*	1.74 × 10^−6^	3.75
*Tgtp2*	8.87 × 10^−17^	−14.41	*Mreg*	3.26 × 10^−6^	3.76
*Gbp3*	3.49 × 10^−49^	−13.68	*Scd2*	1.59 × 10^−43^	3.88
*Ccl20*	7.76 × 10^−5^	−12.90	*Dzip1*	1.14 × 10^−6^	4.05
*Dusp5*	1.04 × 10^−7^	−12.49	*Acacb*	6.33 × 10^−4^	4.18
*Hbegf*	3.01 × 10^−34^	−12.06	*B4gat1*	1.30 × 10^−3^	4.64
*Ifit3*	6.59 × 10^−75^	−11.33	*Acat2*	1.39 × 10^−7^	5.23
**PND8 rat Sertoli APAP + GEN (50 µM)**					
**Downregulated**			**Upregulated**		
Gene	*p*-value	Fold change	Gene	*p*-value	Fold change
*H2ac10*	3.82 × 10^−39^	−8.28	*Pde4d*	2.74 × 10^−12^	2.49
*Pimreg*	9.09 × 10^−4^	−6.10	*Nqo1*	9.70 × 10^−23^	2.54
*E2f8*	9.53 × 10^−8^	−5.40	*Csf2rb*	1.12 × 10^−3^	2.56
*Uhrf1*	6.39 × 10^−26^	−5.24	*Il6*	8.36 × 10^−4^	2.73
*Cdca3*	1.40 × 10^−8^	−5.23	*Eda2r*	5.03 × 10^−26^	2.82
*Pbk*	2.67 × 10^−4^	−4.45	*Cd80*	3.24 × 10^−9^	3.40
*Hist1h2bg*	5.98 × 10^−7^	−4.40	*Cdkn1a*	8.58 × 10^−94^	3.40
*Ckap2l*	5.00 × 10^−10^	−4.20	*Abcb1b*	3.69 × 10^−113^	3.94
*Hist1h2bc*	2.33 × 10^−4^	−4.17	*Star*	3.91 × 10^−179^	4.21
*Cdca5*	5.13 × 10^−6^	−4.14	*Gdf15*	8.82 × 10^−15^	5.88

**Table 3:** Common Functional Pathways Altered in TM4 Cells and PND8 Rat Sertoli Cells by 50 µM APAP + GEN Mixture.

	TM4 Cells			PND8 Rat Sertoli Cells		
Pathway Description	Enrichment Score	*p*-Value	Genes	Enrichment Score	*p*-Value	Genes
Necroptosis	18.7	7.39 × 10^−9^	22	3.4	3.50 × 10^−2^	4
Viral carcinogenesis	10.1	4.01 × 10^−5^	20	9.9	5.17 × 10^−5^	9
Transcriptional misregulation in cancer	13.5	1.36 × 10^−6^	21	7.0	9.35 × 10^−4^	7
MicroRNAs in cancer	6.8	1.10 × 10^−3^	14	4.0	1.80 × 10^−2^	5
p53 signaling pathway	5.9	2.61 × 10^−3^	8	12.5	3.73 × 10^−6^	7
Cellular senescence	6.0	2.47 × 10^−3^	14	10.8	1.95 × 10^−5^	9
TNF signaling pathway	4.5	1.16 × 10^−2^	9	3.9	2.10 × 10^−2^	4
Viral protein interaction with cytokine and cytokine receptor	5.2	5.72 × 10^−3^	8	3.1	4.32 × 10^−2^	3

**Table 4:** Functional pathways uniquely dysregulated by 50 µM APAP + GEN mixture in TM4 cells.

	TM4 Cells		
Pathway Description	Enrichment Score	*p*-Value	Genes
Ribosome biogenesis in eukaryotes	15.8	1.40 × 10^−7^	15
NOD-like receptor signaling pathway	8.2	0.000270	16
AMPK signaling pathway	4.6	0.0100	10
NF-kappa B signaling pathway	4.0	0.0185	8
IL-17 signaling pathway	3.6	0.0280	7
Steroid biosynthesis	3.5	0.0291	3
TGF-beta signaling pathway	3.4	0.0343	7
Cytosolic DNA-sensing pathway	3.0	0.0500	5

**Table 5:** Functional pathways uniquely dysregulated by 50 µM APAP + GEN mixture in PND8 rat Sertoli cells.

	PND8 Rat Sertoli Cells		
Pathway Description	Enrichment Score	*p*-Value	Genes
Foxo signaling pathway	8.6	0.000180	7
Melanoma	3.7	0.0252	3
Glioma	3.6	0.0283	3
PI3K-Akt signaling pathway	3.5	0.0298	7
Pancreatic cancer	3.5	0.0316	3
Rheumatoid arthritis	3.5	0.0316	3
Hematopoietic cell lineage	3.4	0.0351	3
JAK-STAT signaling pathway	3.3	0.0368	4

## Data Availability

The data supporting the study can be found in the article and the Supplementary Materials.

## Supplementary Materials

The following supporting information can be downloaded at: https://www.mdpi.com/article/10.3390/cells12131804/s1. Figure S1. Workflow Diagram of immature PND8 Sertoli cell isolation, and evaluation of cell purity. Figure S2. Diagrams of estrogen receptor (A) and eicosanoids (B) signaling pathways in TM4 cells treated with 50 µM APAP-GEN mixture.
