# Supplementary material for: Dysregulation of Immature Sertoli Cell Functions by Exposure to Acetaminophen and Genistein in Rodent Cell Models
Source: Cells. 2023 Jul 7;12(13):1804. doi: 10.3390/cells12131804 (PMC10340629; doi:10.3390/cells12131804)
Supplement: Supplementary file 1 [file cells-12-01804-s001.zip › cells-2365170-supplementary.pdf]

Supplemental figures S1 and S2

Dysregulation of immature Sertoli cell functions by exposure to acetaminophen and genistein in rodent cell models

Maia Corpuz-Hilsabeck, Nicole Mohajer, Martine Culty

Supplemental Figure S1. Workflow Diagram of immature PND8 Sertoli cell isolation, and evaluation of cell purity. (A) Diagram of the main steps for isolated immature Sertoli cell preparations from PND8 rat testes. Details are provided in the Method section. The collagenase-based step dissociates interstitial cells (Leydig cells, blood vessel, hematopoietic cells, fibroblasts) and allows their removal. The trypsin EDTA-based digestion dissociates cells from the seminiferous tubules (Sertoli, peritubular myoid and germ cells). After overnight cell suspension plating to allow Sertoli and myoid cells adhesion, floating germ cells are removed. Sertoli cells are plated in wells and treated as needed for subsequent mRNA and protein analyses. **(B)** Evaluation of Sertoli cell purity by immunofluorescence (IF) analysis. Pictures of IF analysis were taken and cell counts were performed for Sertoli cell population labeled with anti-Vimentin antibody (red) (left), while myoid cells were identified using anti-α-SMA antibody (red) (right), and total cell numbers were counted based on DAPI (blue) nuclear staining. Using Fiji is Just ImageJ (FIJI) software, Sertoli and myoid cells were quantified, and their proportion expressed as percent of total cells numbers counted using DAPI (blue). Representative samples are shown.

Supplemental Figure S2. Diagrams of estrogen receptor (A) and eicosanoids (B) signaling pathways in TM4 cells treated with 50µM APAP-GEN mixture. The diagrams obtained by Ingenuity Pathway. Red: upregulated genes; Green: downregulated genes; Orange: predicted activation; Blue: predicted inhibition. Darker colors indicating stronger datasets. Yellow line: findings inconsistent with state of downstream molecule; Grey line: Effect not predicted.

Supplemental Figure S1

A

1. **Testes collection and decapsulation** from 10 PND8 rats
2. **Dissociation of interstitial cells** and release of seminiferous tubules:
   Collagenase + Hyaluronidase + Dnase I in 37˚C shaking water bath
3. **Sedimentation of seminiferous tubules** — **Remove supernatant** with interstitial cells
4. **Dissociation of seminiferous tubules:** Trypsin-EDTA + Dnase I in 37˚C shaking water bath + Mechanical Dissociation
5. **Sedimentation of tubule fragments -** If large fragments, repeat trypsinization for 5-10 min
6. **Dilute cell supernatant (1:1) with 10% FBS** in RPMI 1640 with antibiotics
   Suspension of Sertoli, peritubular myoid & germ cells + tubule fragments
7. **Filtration** on 45 µm insert (remove fragments)
8. **Centrifugation** (800g; 5 min) - **Resuspended cell pellet** in medium + 5% CS-FBS
9. **Overnight Differential plating:** Sertoli cell adhesion (~30% myoid cells)
10. **Remove supernatant** containing non-adherent floating germ cells
11. **Wash twice** adherent cell layer with PBS
12. **Detach adherent Sertoli cells** with trypsin-EDTA – Then add medium + 10 % CS-FBS
13. **Centrifugation** (800g; 5 min) - **Resuspended cell pellet in medium + 10 % CS-FBS**
14. **Determine Sertoli cell count and purity**
15. **Plate cells in wells as needed** for experiments. Wait 5 hours before adding treatments
16. **Treatments** (vehicle, APAP, GEN alone or mixed) and **endpoints to analyze**

Cell viability/proliferation | **qPCR/RNA-seq analyses** | **Immunofluorescence**

B

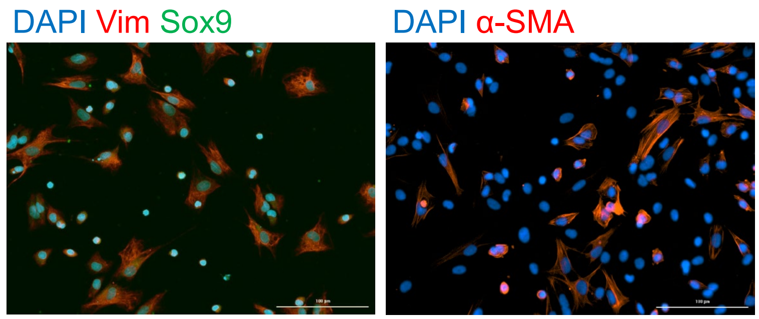

Supplemental Figure S2

A

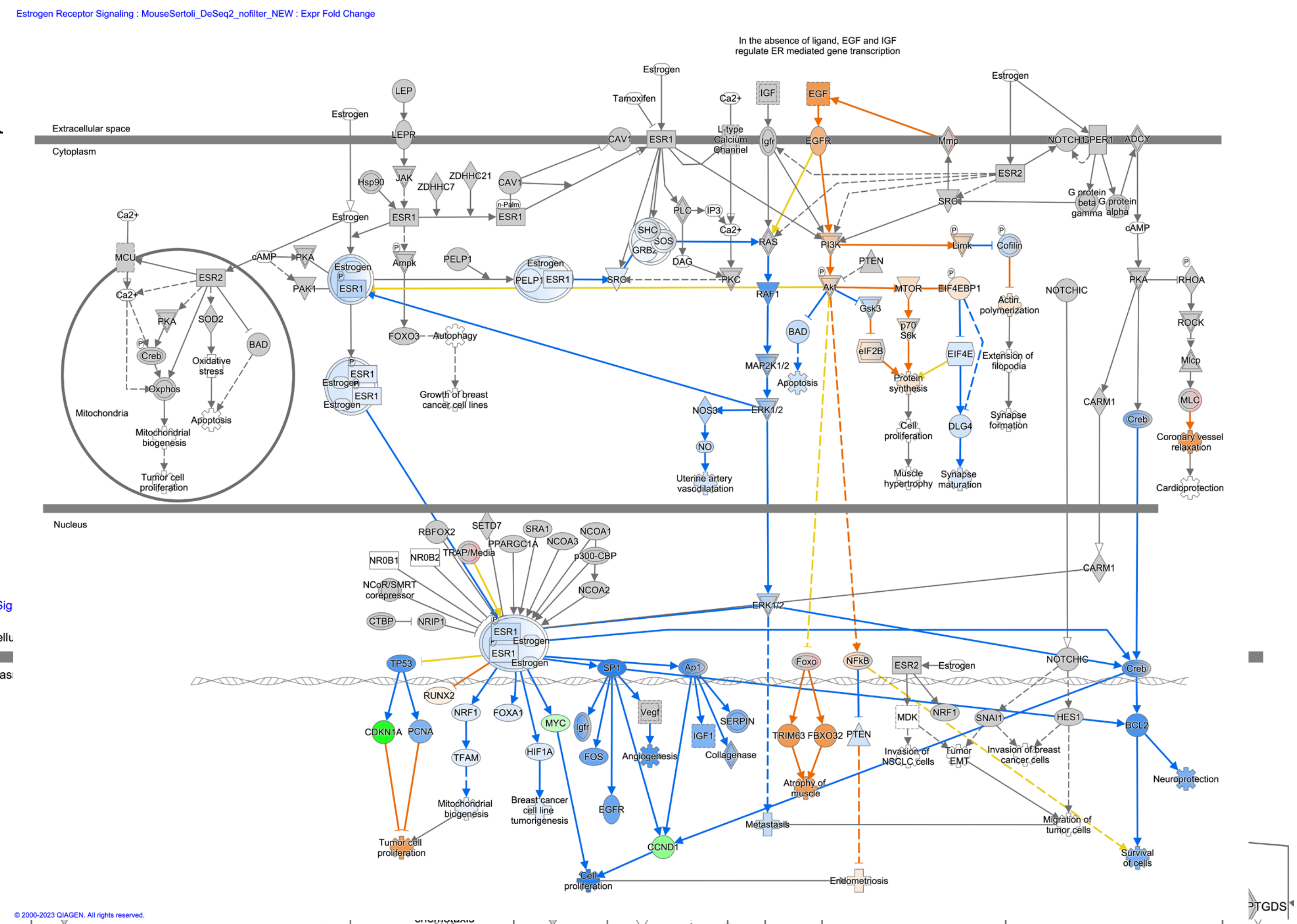

B

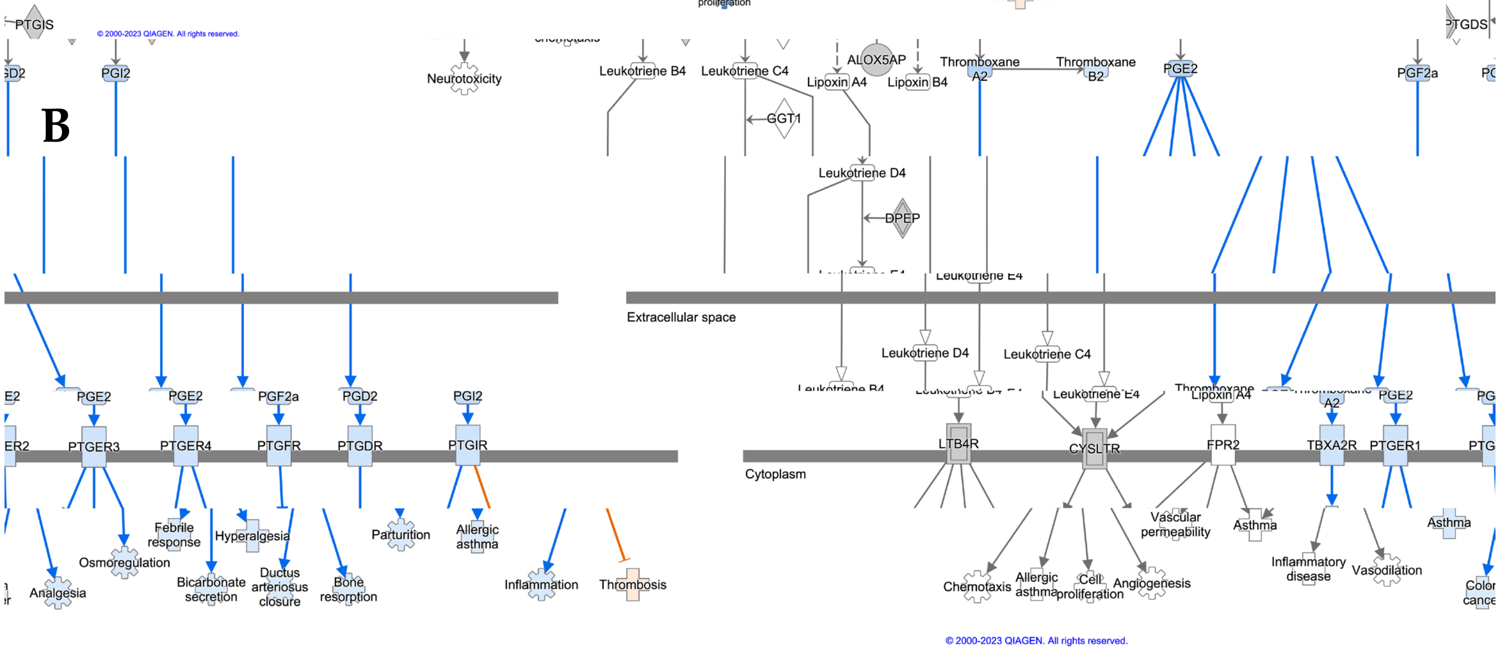

TM4 Sertoli cell line DEGs

Gene ID	Gene name	P-value (A	FDR step u	Ratio (A50	Fold chang	LSMean(A	LSMean(V	P-value (G	FDR step u	Ratio (G50	Fold chang	LSMean(G	LSMean(V	P-value (A
Ereg	Ereg	2.36E-11	9.13E-08	2.04E-01	-4.90	2.88E+01	1.41E+02	6.71E-35	8.66E-32	6.75E-02	-1.48E+01	9.53E+00	1.41E+02	2.19E-31
Hbegf	Hbegf	8.14E-10	1.10E-06	2.97E-01	-3.37	5.05E+01	1.70E+02	1.64E-42	3.18E-39	8.28E-02	-1.21E+01	1.41E+01	1.70E+02	3.01E-34
Lif	Lif	1.39E-07	1.10E-04	3.37E-01	-2.96	4.76E+01	1.41E+02	2.29E-22	1.18E-19	1.63E-01	-6.14E+00	2.30E+01	1.41E+02	3.07E-26
Cth	Cth	1.14E-11	5.15E-08	3.52E-01	-2.84	9.36E+01	2.66E+02	6.66E-16	2.15E-13	3.30E-01	-3.03E+00	8.77E+01	2.66E+02	7.84E-21
H1f1	H1f1	4.41E-18	5.98E-14	3.92E-01	-2.55	2.59E+02	6.60E+02	2.19E-74	1.19E-70	1.69E-01	-5.92E+00	1.11E+02	6.60E+02	6.31E-66
H1f0	H1f0	3.34E-21	9.06E-17	3.93E-01	-2.54	4.10E+02	1.04E+03	6.98E-18	2.56E-15	4.68E-01	-2.14E+00	4.88E+02	1.04E+03	6.18E-17
Il1rl1	Il1rl1	5.63E-08	5.26E-05	3.98E-01	-2.51	8.91E+01	2.24E+02	1.63E-36	2.45E-33	1.43E-01	-7.01E+00	3.20E+01	2.24E+02	1.39E-53
Mtmr10	Mtmr10	4.93E-05	1.50E-02	3.99E-01	-2.51	3.79E+01	9.50E+01	1.39E-09	2.04E-07	2.92E-01	-3.42E+00	2.78E+01	9.50E+01	2.63E-10
H4f16	H4f16	4.61E-10	7.81E-07	4.18E-01	-2.39	1.44E+02	3.44E+02	9.40E-20	4.04E-17	3.20E-01	-3.13E+00	1.10E+02	3.44E+02	6.42E-15
Prkg2	Prkg2	2.33E-10	4.52E-07	4.26E-01	-2.35	1.91E+02	4.48E+02	9.86E-55	2.67E-51	1.49E-01	-6.70E+00	6.68E+01	4.48E+02	9.85E-53
Mphosph1	Mphosph1	5.56E-10	8.87E-07	4.32E-01	-2.31	1.45E+02	3.35E+02	4.06E-21	1.90E-18	3.19E-01	-3.14E+00	1.07E+02	3.35E+02	1.18E-21
Ifi211	Ifi211	5.95E-08	5.38E-05	4.35E-01	-2.30	1.20E+02	2.76E+02	2.15E-35	2.91E-32	1.78E-01	-5.61E+00	4.92E+01	2.76E+02	3.77E-39
Chka	Chka	3.00E-05	9.98E-03	4.42E-01	-2.26	6.89E+01	1.56E+02	3.08E-15	9.19E-13	2.49E-01	-4.01E+00	3.89E+01	1.56E+02	7.50E-15
Timm8a1	Timm8a1	3.24E-07	2.25E-04	4.44E-01	-2.25	1.11E+02	2.50E+02	2.38E-22	1.19E-19	2.49E-01	-4.02E+00	6.23E+01	2.50E+02	2.29E-21
Errfi1	Errfi1	3.54E-08	3.55E-05	4.50E-01	-2.22	1.49E+02	3.31E+02	3.65E-30	3.66E-27	2.26E-01	-4.43E+00	7.46E+01	3.31E+02	7.21E-47
Cnbp	Cnbp	2.56E-17	2.31E-13	4.71E-01	-2.12	6.20E+02	1.32E+03	1.27E-32	1.49E-29	3.88E-01	-2.58E+00	5.11E+02	1.32E+03	5.41E-35
Crls1	Crls1	2.69E-05	9.34E-03	4.72E-01	-2.12	6.98E+01	1.48E+02	6.32E-05	3.21E-03	5.28E-01	-1.89E+00	7.82E+01	1.48E+02	1.24E-07
H3c7	H3c7	1.36E-04	3.12E-02	4.73E-01	-2.12	8.27E+01	1.75E+02	3.49E-15	1.03E-12	2.49E-01	-4.02E+00	4.35E+01	1.75E+02	9.41E-12
Ddx10	Ddx10	2.34E-05	8.34E-03	4.74E-01	-2.11	7.03E+01	1.48E+02	1.99E-08	2.46E-06	4.12E-01	-2.43E+00	6.11E+01	1.48E+02	4.09E-11
H4c2	H4c2	8.10E-06	3.72E-03	4.74E-01	-2.11	1.03E+02	2.17E+02	2.28E-09	3.24E-07	4.09E-01	-2.44E+00	8.87E+01	2.17E+02	3.00E-06
Epha2	Epha2	9.78E-05	2.52E-02	4.76E-01	-2.10	8.64E+01	1.82E+02	2.64E-24	1.55E-21	1.73E-01	-5.79E+00	3.14E+01	1.82E+02	3.22E-26
H3c2	H3c2	2.36E-07	1.73E-04	4.79E-01	-2.09	1.61E+02	3.36E+02	1.01E-24	6.22E-22	2.69E-01	-3.72E+00	9.03E+01	3.36E+02	6.96E-19
Ostc	Ostc	6.28E-06	2.99E-03	4.83E-01	-2.07	7.47E+01	1.55E+02	4.48E-02	5.55E-01	7.50E-01	-1.33E+00	1.16E+02	1.55E+02	2.09E-05
H1f2	H1f2	6.10E-12	3.31E-08	4.88E-01	-2.05	4.70E+02	9.63E+02	6.71E-27	5.35E-24	3.67E-01	-2.73E+00	3.53E+02	9.63E+02	1.69E-39
Arap2	Arap2	2.30E-05	8.34E-03	4.97E-01	-2.01	7.90E+01	1.59E+02	1.51E-06	1.26E-04	4.92E-01	-2.03E+00	7.82E+01	1.59E+02	3.32E-09
Dag1	Dag1	1.13E-04	2.80E-02	2.09E+00	2.09	9.05E+01	4.33E+01	8.66E-07	7.70E-05	2.32E+00	2.32E+00	1.00E+02	4.33E+01	4.49E-08
Rpa1	Rpa1	1.29E-04	3.03E-02	2.11E+00	2.11	1.00E+02	4.74E+01	1.51E-01	9.96E-01	1.29E+00	1.29E+00	6.09E+01	4.74E+01	6.90E-03
Ubc	Ubc	2.08E-07	1.56E-04	2.20E+00	2.20	3.19E+02	1.45E+02	5.53E-04	1.98E-02	1.60E+00	1.60E+00	2.32E+02	1.45E+02	4.86E-07
Mki67	Mki67	1.08E-14	7.34E-11	2.29E+00	2.29	5.57E+02	2.44E+02	1.39E-05	8.66E-04	1.52E+00	1.52E+00	3.70E+02	2.44E+02	8.78E-15
Kif20a	Kif20a	4.78E-06	2.49E-03	2.32E+00	2.32	1.04E+02	4.49E+01	1.71E-07	1.75E-05	2.37E+00	2.37E+00	1.06E+02	4.49E+01	1.43E-08
Ckap2l	Ckap2l	2.18E-06	1.31E-03	2.43E+00	2.43	1.01E+02	4.15E+01	1.45E-03	4.37E-02	1.71E+00	1.71E+00	7.10E+01	4.15E+01	9.00E-07
Acat2	Acat2	1.76E-04	3.66E-02	3.28E+00	3.28	2.30E+01	7.02E+00	3.36E-09	4.64E-07	5.35E+00	5.35E+00	3.75E+01	7.02E+00	1.39E-07
Mettl26	Mettl26	1.93E-05	7.81E-03	8.46E+00	8.46	4.05E+01	4.78E+00	3.78E-06	2.78E-04	7.98E+00	7.98E+00	3.82E+01	4.78E+00	3.11E-07

Gm23971	Gm23971	9.09E-05	2.41E-02	1.30E+01	12.97	2.95E+01	2.27E+00	1.17E-06	1.01E-04	1.75E+01	1.75E+01	3.98E+01	2.27E+00	4.80E-08
n-R5s56	n-R5s56	1.06E-04	2.69E-02	3.39E+01	33.95	1.15E+01	3.37E-01	2.84E-05	1.61E-03	3.24E+01	3.24E+01	1.09E+01	3.37E-01	6.91E-06
n-R5s2	n-R5s2	1.87E-04	3.81E-02	7.03E+01	70.26	8.53E+00	1.21E-01	1.21E-04	5.52E-03	5.82E+01	5.82E+01	7.07E+00	1.21E-01	1.30E-04
Gm23650	Gm23650	1.39E-04	3.13E-02	7.17E+01	71.69	8.70E+00	1.21E-01	1.22E-04	5.57E-03	5.49E+01	5.49E+01	6.67E+00	1.21E-01	2.74E-05
Gm24245	Gm24245	5.96E-05	1.70E-02	8.81E+01	88.14	1.07E+01	1.21E-01	9.63E-04	3.12E-02	3.11E+01	3.11E+01	3.77E+00	1.21E-01	2.86E-05
Rnu2-10	Rnu2-10	1.81E-04	3.73E-02	1.21E+02	121.21	1.48E+01	1.22E-01	1.58E-05	9.68E-04	1.64E+02	1.64E+02	2.00E+01	1.22E-01	8.76E-06
Gm24601	Gm24601	1.25E-04	3.03E-02	1.30E+02	130.30	1.58E+01	1.21E-01	2.48E-05	1.43E-03	1.39E+02	1.39E+02	1.69E+01	1.21E-01	1.14E-05
n-R5s106	n-R5s106	2.33E-05	8.34E-03	2.47E+02	246.86	3.00E+01	1.22E-01	1.76E-06	1.43E-04	3.08E+02	3.08E+02	3.74E+01	1.22E-01	1.26E-06
n-R5s130	n-R5s130	2.33E-05	8.34E-03	2.47E+02	246.86	3.00E+01	1.22E-01	1.76E-06	1.43E-04	3.08E+02	3.08E+02	3.74E+01	1.22E-01	1.26E-06
n-R5-8s1	n-R5-8s1	2.08E-05	8.28E-03	2.50E+02	250.17	4.15E+01	1.66E-01	1.25E-06	1.07E-04	3.13E+02	3.13E+02	5.20E+01	1.66E-01	1.43E-06

FDR step u	Ratio (AG5	Fold chang	LSMean(A	LSMean(Vehicle) (AG50 vs Vehicle)
2.60E-28	5.61E-02	-1.78E+01	7.92E+00	1.41E+02
4.08E-31	8.29E-02	-1.21E+01	1.41E+01	1.70E+02
2.52E-23	1.06E-01	-9.41E+00	1.50E+01	1.41E+02
4.34E-18	2.36E-01	-4.23E+00	6.27E+01	2.66E+02
3.42E-62	1.54E-01	-6.51E+00	1.01E+02	6.60E+02
2.66E-14	4.39E-01	-2.28E+00	4.57E+02	1.04E+03
5.36E-50	6.40E-02	-1.56E+01	1.43E+01	2.24E+02
5.27E-08	2.37E-01	-4.22E+00	2.25E+01	9.50E+01
2.18E-12	3.36E-01	-2.98E+00	1.16E+02	3.44E+02
3.34E-49	1.23E-01	-8.13E+00	5.50E+01	4.48E+02
7.30E-19	2.73E-01	-3.66E+00	9.14E+01	3.35E+02
6.01E-36	1.29E-01	-7.77E+00	3.55E+01	2.76E+02
2.51E-12	2.15E-01	-4.64E+00	3.36E+01	1.56E+02
1.38E-18	2.19E-01	-4.58E+00	5.47E+01	2.50E+02
1.96E-43	1.19E-01	-8.40E+00	3.94E+01	3.31E+02
8.15E-32	3.33E-01	-3.00E+00	4.38E+02	1.32E+03
1.47E-05	3.88E-01	-2.58E+00	5.75E+01	1.48E+02
2.45E-09	2.60E-01	-3.84E+00	4.56E+01	1.75E+02
9.40E-09	3.10E-01	-3.22E+00	4.61E+01	1.48E+02
2.40E-04	4.59E-01	-2.18E+00	9.94E+01	2.17E+02
2.57E-23	1.27E-01	-7.86E+00	2.31E+01	1.82E+02
3.63E-16	2.81E-01	-3.56E+00	9.44E+01	3.36E+02
1.33E-03	5.05E-01	-1.98E+00	7.81E+01	1.55E+02
2.86E-36	2.52E-01	-3.97E+00	2.43E+02	9.63E+02
5.67E-07	3.76E-01	-2.66E+00	5.98E+01	1.59E+02
5.86E-06	2.83E+00	2.83E+00	1.23E+02	4.33E+01
1.46E-01	1.69E+00	1.69E+00	8.03E+01	4.74E+01
4.94E-05	2.15E+00	2.15E+00	3.11E+02	1.45E+02
2.90E-12	2.29E+00	2.29E+00	5.59E+02	2.44E+02
2.12E-06	2.83E+00	2.83E+00	1.27E+02	4.49E+01
8.65E-05	2.51E+00	2.51E+00	1.04E+02	4.15E+01
1.60E-05	5.23E+00	5.23E+00	3.68E+01	7.02E+00
3.25E-05	1.29E+01	1.29E+01	6.15E+01	4.78E+00

6.20E-06	3.55E+01	3.55E+01	8.05E+01	2.27E+00
5.01E-04	5.93E+01	5.93E+01	2.00E+01	3.37E-01
6.03E-03	7.76E+01	7.76E+01	9.42E+00	1.21E-01
1.68E-03	1.09E+02	1.09E+02	1.33E+01	1.21E-01
1.72E-03	1.06E+02	1.06E+02	1.29E+01	1.21E-01
6.23E-04	2.97E+02	2.97E+02	3.62E+01	1.22E-01
7.76E-04	2.61E+02	2.61E+02	3.18E+01	1.21E-01
1.17E-04	5.49E+02	5.49E+02	6.67E+01	1.22E-01
1.17E-04	5.49E+02	5.49E+02	6.67E+01	1.22E-01
1.29E-04	5.20E+02	5.20E+02	8.62E+01	1.66E-01

PND8 rat Sertoli cells DEGs

Gene ID	Gene name	P-value (A	FDR step u	Ratio (A50	Fold chang	LSMean(A	LSMean(V	P-value (G	FDR step u	Ratio (G50
Ttll13	Ttll13	2.17E-01	1.00E+00	3.85E-01	-2.59E+00	5.65E-01	1.47E+00	3.62E-05	1.60E-03	3.28E-02
ENSRNOC	---	8.31E-01	1.00E+00	8.23E-01	-1.21E+00	9.47E-01	1.15E+00	1.72E-03	4.27E-02	5.87E-02
Depdc1b	Depdc1b	8.78E-01	1.00E+00	1.10E+00	1.10E+00	8.41E-01	7.61E-01	7.54E-04	2.16E-02	1.00E-01
H2ac10	H2ac10	7.34E-01	1.00E+00	1.06E+00	1.06E+00	2.72E+01	2.57E+01	1.39E-37	1.54E-34	1.27E-01
E2f8	E2f8	6.88E-01	1.00E+00	8.74E-01	-1.14E+00	4.12E+00	4.72E+00	4.22E-08	3.82E-06	1.76E-01
Sgo2	Sgo2	7.69E-01	1.00E+00	8.97E-01	-1.12E+00	5.20E+00	5.80E+00	2.73E-06	1.62E-04	2.00E-01
Cenpq	Cenpq	8.79E-01	1.00E+00	1.08E+00	1.08E+00	1.56E+00	1.44E+00	1.56E-03	3.95E-02	2.02E-01
Pbk	Pbk	7.98E-01	1.00E+00	1.12E+00	1.12E+00	2.50E+00	2.24E+00	1.09E-04	4.15E-03	2.03E-01
Cdca3	Cdca3	9.67E-01	1.00E+00	1.01E+00	1.01E+00	3.77E+00	3.73E+00	8.23E-08	7.08E-06	2.12E-01
Depdc1	Depdc1	8.94E-01	1.00E+00	9.45E-01	-1.06E+00	2.21E+00	2.33E+00	1.17E-04	4.38E-03	2.12E-01
Hist1h2bc	Hist1h2bc	8.76E-01	1.00E+00	9.37E-01	-1.07E+00	2.96E+00	3.16E+00	7.92E-05	3.13E-03	2.14E-01
Uhrf1	Uhrf1	5.41E-01	1.00E+00	1.11E+00	1.11E+00	2.00E+01	1.80E+01	1.10E-21	5.61E-19	2.25E-01
Kif4a	Kif4a	5.90E-01	1.00E+00	1.13E+00	1.13E+00	6.94E+00	6.14E+00	1.71E-11	2.56E-09	2.30E-01
Racgap1	Racgap1	7.96E-01	1.00E+00	1.07E+00	1.07E+00	8.47E+00	7.92E+00	4.01E-09	4.28E-07	2.46E-01
Tk1	Tk1	9.95E-01	1.00E+00	1.00E+00	1.00E+00	8.64E+00	8.63E+00	1.50E-12	2.63E-10	2.50E-01
Nr0b1	Nr0b1	6.76E-01	1.00E+00	9.22E-01	-1.08E+00	8.32E+00	9.02E+00	7.62E-14	1.59E-11	2.55E-01
Esco2	Esco2	9.47E-01	1.00E+00	1.03E+00	1.03E+00	2.95E+00	2.87E+00	4.65E-04	1.45E-02	2.62E-01
Clspn	Clspn	6.52E-01	1.00E+00	1.12E+00	1.12E+00	1.23E+01	1.10E+01	2.28E-09	2.51E-07	2.65E-01
Aunip	Aunip	7.81E-01	1.00E+00	8.90E-01	-1.12E+00	1.49E+00	1.67E+00	9.19E-04	2.55E-02	2.70E-01
Cit	Cit	9.27E-01	1.00E+00	1.03E+00	1.03E+00	5.18E+00	5.05E+00	2.05E-06	1.26E-04	2.73E-01
Iqgap3	Iqgap3	5.04E-01	1.00E+00	1.25E+00	1.25E+00	6.29E+00	5.03E+00	2.77E-05	1.25E-03	2.74E-01
Cenpf	Cenpf	8.62E-01	1.00E+00	9.77E-01	-1.02E+00	3.44E+01	3.52E+01	9.11E-26	6.86E-23	2.75E-01
Cilp	Cilp	8.58E-02	1.00E+00	7.03E-01	-1.42E+00	1.65E+01	2.35E+01	6.00E-12	9.49E-10	2.78E-01
Ttk	Ttk	8.29E-01	1.00E+00	1.07E+00	1.07E+00	4.55E+00	4.25E+00	1.53E-05	7.54E-04	2.79E-01
Ckap2l	Ckap2l	6.34E-01	1.00E+00	1.12E+00	1.12E+00	8.12E+00	7.22E+00	3.25E-08	3.08E-06	2.82E-01
Cep55	Cep55	7.21E-01	1.00E+00	1.10E+00	1.10E+00	4.86E+00	4.40E+00	1.58E-06	9.93E-05	2.83E-01
E2f7	E2f7	6.45E-01	1.00E+00	1.16E+00	1.16E+00	3.01E+00	2.60E+00	3.40E-05	1.51E-03	2.83E-01
Cdc6	Cdc6	8.76E-01	1.00E+00	1.06E+00	1.06E+00	3.19E+00	3.01E+00	1.73E-04	6.12E-03	2.86E-01
Mki67	Mki67	5.98E-01	1.00E+00	1.06E+00	1.06E+00	7.59E+01	7.17E+01	2.06E-36	2.15E-33	2.86E-01
Ccna2	Ccna2	4.75E-01	1.00E+00	1.16E+00	1.16E+00	1.21E+01	1.04E+01	1.05E-10	1.41E-08	2.89E-01
Cdca5	Cdca5	5.75E-01	1.00E+00	8.32E-01	-1.20E+00	2.30E+00	2.76E+00	5.86E-05	2.43E-03	2.91E-01
Knstrn	Knstrn	9.56E-01	1.00E+00	1.02E+00	1.02E+00	2.20E+00	2.16E+00	1.01E-03	2.74E-02	2.91E-01
Spag5	Spag5	5.37E-01	1.00E+00	1.15E+00	1.15E+00	7.93E+00	6.90E+00	5.42E-09	5.58E-07	2.91E-01
H3c1	H3c1	6.13E-01	1.00E+00	1.23E+00	1.23E+00	3.35E+00	2.71E+00	1.46E-03	3.73E-02	2.92E-01
Kif14	Kif14	8.70E-01	1.00E+00	1.06E+00	1.06E+00	4.33E+00	4.11E+00	6.73E-05	2.73E-03	2.97E-01
Hist1h2bg	Hist1h2bg	9.14E-01	1.00E+00	9.67E-01	-1.03E+00	3.03E+00	3.13E+00	3.19E-05	1.42E-03	2.98E-01
Kif11	Kif11	6.52E-01	1.00E+00	1.10E+00	1.10E+00	1.23E+01	1.13E+01	1.19E-10	1.59E-08	2.99E-01
Mcm5	Mcm5	6.77E-01	1.00E+00	1.07E+00	1.07E+00	1.92E+01	1.80E+01	5.75E-17	1.75E-14	2.99E-01
Nusap1	Nusap1	3.79E-01	1.00E+00	1.29E+00	1.29E+00	5.04E+00	3.91E+00	9.28E-06	4.80E-04	3.00E-01
Shcbp1	Shcbp1	4.94E-01	1.00E+00	1.21E+00	1.21E+00	6.56E+00	5.45E+00	2.54E-06	1.52E-04	3.02E-01
Ect2	Ect2	8.30E-01	1.00E+00	1.06E+00	1.06E+00	7.57E+00	7.17E+00	4.44E-07	3.40E-05	3.03E-01
Plk1	Plk1	5.99E-01	1.00E+00	1.12E+00	1.12E+00	6.74E+00	6.00E+00	1.06E-08	1.07E-06	3.04E-01
Cenpe	Cenpe	6.98E-01	1.00E+00	1.05E+00	1.05E+00	2.80E+01	2.67E+01	1.14E-25	8.22E-23	3.04E-01
Hist1h2ac	Hist1h2ac	5.12E-01	1.00E+00	1.18E+00	1.18E+00	5.83E+00	4.93E+00	7.30E-07	5.27E-05	3.06E-01
Ccnb1	Ccnb1	7.19E-01	1.00E+00	1.12E+00	1.12E+00	6.90E+00	6.16E+00	4.51E-05	1.91E-03	3.06E-01
Cdk1	Cdk1	9.44E-01	1.00E+00	1.02E+00	1.02E+00	5.84E+00	5.72E+00	1.44E-05	7.12E-04	3.11E-01
Fam83d	Fam83d	7.57E-01	1.00E+00	9.12E-01	-1.10E+00	3.62E+00	3.97E+00	2.58E-05	1.18E-03	3.11E-01

Nuf2	Nuf2	9.12E-01	1.00E+00	1.03E+00	1.03E+00	7.30E+00	7.10E+00	7.55E-07	5.40E-05	3.11E-01
Cdca2	Cdca2	7.45E-01	1.00E+00	1.09E+00	1.09E+00	6.14E+00	5.65E+00	1.19E-06	7.92E-05	3.13E-01
Bub1b	Bub1b	4.31E-01	1.00E+00	1.21E+00	1.21E+00	9.63E+00	7.95E+00	2.56E-07	2.01E-05	3.14E-01
Kifc1	Kifc1	9.30E-01	1.00E+00	1.02E+00	1.02E+00	7.72E+00	7.56E+00	4.77E-07	3.62E-05	3.16E-01
Kif2c	Kif2c	7.75E-01	1.00E+00	1.08E+00	1.08E+00	4.16E+00	3.85E+00	5.77E-06	3.17E-04	3.16E-01
Tacc3	Tacc3	8.67E-01	1.00E+00	1.04E+00	1.04E+00	1.02E+01	9.80E+00	3.44E-07	2.69E-05	3.18E-01
Top2a	Top2a	5.80E-02	1.00E+00	1.22E+00	1.22E+00	3.88E+01	3.17E+01	7.62E-30	6.24E-27	3.25E-01
Anln	Anln	3.66E-01	1.00E+00	1.17E+00	1.17E+00	1.42E+01	1.21E+01	3.24E-12	5.30E-10	3.26E-01
ENSRNOC	---	7.53E-01	1.00E+00	1.12E+00	1.12E+00	8.91E+00	7.99E+00	3.72E-04	1.20E-02	3.26E-01
Mfap5	Mfap5	7.48E-01	1.00E+00	9.01E-01	-1.11E+00	2.24E+00	2.49E+00	2.32E-04	7.87E-03	3.27E-01
Nek2l1	Nek2l1	7.04E-01	1.00E+00	1.14E+00	1.14E+00	3.67E+00	3.23E+00	4.02E-04	1.28E-02	3.28E-01
Cenpu	Cenpu	9.39E-01	1.00E+00	9.74E-01	-1.03E+00	3.52E+00	3.62E+00	5.45E-04	1.65E-02	3.29E-01
Ndc80	Ndc80	4.81E-01	1.00E+00	1.17E+00	1.17E+00	9.22E+00	7.91E+00	5.60E-08	4.96E-06	3.31E-01
Brip1	Brip1	7.17E-01	1.00E+00	1.09E+00	1.09E+00	5.68E+00	5.22E+00	5.00E-07	3.75E-05	3.32E-01
Kif20a	Kif20a	8.43E-01	1.00E+00	9.53E-01	-1.05E+00	9.59E+00	1.01E+01	8.47E-07	5.92E-05	3.32E-01
Prc1	Prc1	9.99E-01	1.00E+00	1.00E+00	-1.00E+00	1.85E+01	1.85E+01	5.46E-12	8.79E-10	3.35E-01
Kntc1	Kntc1	3.30E-01	1.00E+00	1.21E+00	1.21E+00	1.32E+01	1.09E+01	9.58E-10	1.13E-07	3.36E-01
Bard1	Bard1	7.35E-01	1.00E+00	1.09E+00	1.09E+00	4.94E+00	4.52E+00	7.93E-06	4.16E-04	3.37E-01
Cenpa	Cenpa	9.29E-01	1.00E+00	9.70E-01	-1.03E+00	4.47E+00	4.61E+00	4.84E-04	1.49E-02	3.41E-01
H1f1	H1f1	3.03E-01	1.00E+00	1.16E+00	1.16E+00	2.41E+01	2.07E+01	1.79E-15	4.54E-13	3.44E-01
ENSRNOC	---	3.98E-01	1.00E+00	1.18E+00	1.18E+00	1.90E+01	1.61E+01	5.64E-09	5.77E-07	3.55E-01
Diaph3	Diaph3	9.76E-01	1.00E+00	9.95E-01	-1.01E+00	1.69E+01	1.70E+01	5.92E-11	8.20E-09	3.55E-01
Hist2h3c2	Hist2h3c2	2.85E-01	1.00E+00	1.15E+00	1.15E+00	1.31E+02	1.14E+02	5.82E-18	2.03E-15	3.57E-01
Ncapg	Ncapg	5.25E-01	1.00E+00	1.14E+00	1.14E+00	8.83E+00	7.75E+00	7.27E-08	6.34E-06	3.57E-01
Dmpk	Dmpk	4.69E-01	1.00E+00	8.20E-01	-1.22E+00	5.15E+00	6.29E+00	4.76E-05	2.01E-03	3.60E-01
Cenph	Cenph	7.68E-01	1.00E+00	1.10E+00	1.10E+00	3.23E+00	2.94E+00	5.08E-04	1.55E-02	3.60E-01
Tcf19	Tcf19	9.01E-01	1.00E+00	9.70E-01	-1.03E+00	7.59E+00	7.82E+00	5.63E-06	3.11E-04	3.61E-01
Melk	Melk	4.93E-01	1.00E+00	1.20E+00	1.20E+00	4.57E+00	3.80E+00	7.79E-05	3.09E-03	3.69E-01
Tpx2	Tpx2	3.76E-01	1.00E+00	1.12E+00	1.12E+00	2.10E+01	1.88E+01	1.75E-17	5.49E-15	3.70E-01
Hist1h2ak	Hist1h2ak	5.26E-01	1.00E+00	1.21E+00	1.21E+00	7.24E+00	5.98E+00	3.77E-04	1.22E-02	3.74E-01
Mastl	Mastl	3.21E-01	1.00E+00	1.32E+00	1.32E+00	4.37E+00	3.32E+00	2.22E-04	7.61E-03	3.80E-01
Spc25	Spc25	9.41E-01	1.00E+00	1.02E+00	1.02E+00	2.68E+00	2.62E+00	1.24E-03	3.26E-02	3.86E-01
Kif23	Kif23	8.53E-01	1.00E+00	9.61E-01	-1.04E+00	1.10E+01	1.15E+01	9.64E-07	6.60E-05	3.88E-01
ENSRNOC	---	6.51E-01	1.00E+00	8.64E-01	-1.16E+00	2.62E+00	3.04E+00	1.61E-03	4.04E-02	3.90E-01
Kif18b	Kif18b	7.90E-01	1.00E+00	1.06E+00	1.06E+00	6.61E+00	6.26E+00	9.96E-07	6.77E-05	3.90E-01
Tll1	Tll1	1.44E-01	1.00E+00	7.16E-01	-1.40E+00	3.28E+00	4.58E+00	1.09E-05	5.57E-04	3.96E-01
Sgo1	Sgo1	4.72E-01	1.00E+00	1.23E+00	1.23E+00	4.38E+00	3.56E+00	5.94E-04	1.77E-02	3.96E-01
Ncaph	Ncaph	5.13E-01	1.00E+00	1.14E+00	1.14E+00	8.25E+00	7.27E+00	3.07E-07	2.41E-05	3.97E-01
Ticrr	Ticrr	7.90E-01	1.00E+00	1.08E+00	1.08E+00	5.01E+00	4.64E+00	4.76E-04	1.47E-02	4.02E-01
Mcm10	Mcm10	5.83E-01	1.00E+00	1.13E+00	1.13E+00	6.88E+00	6.07E+00	1.71E-05	8.27E-04	4.04E-01
Kif20b	Kif20b	6.12E-01	1.00E+00	1.11E+00	1.11E+00	1.21E+01	1.09E+01	1.40E-06	8.97E-05	4.06E-01
Arhgap11a	Arhgap11a	4.22E-01	1.00E+00	1.11E+00	1.11E+00	1.48E+01	1.33E+01	6.51E-13	1.18E-10	4.06E-01
C1qtnf1	C1qtnf1	1.31E-01	1.00E+00	7.40E-01	-1.35E+00	6.02E+00	8.14E+00	8.48E-07	5.92E-05	4.08E-01
Aspm	Aspm	8.15E-01	1.00E+00	1.03E+00	1.03E+00	1.90E+01	1.84E+01	1.84E-12	3.18E-10	4.10E-01
Mybl2	Mybl2	7.65E-01	1.00E+00	1.06E+00	1.06E+00	6.49E+00	6.09E+00	5.29E-06	2.93E-04	4.12E-01
Aurkb	Aurkb	9.77E-01	1.00E+00	9.93E-01	-1.01E+00	5.87E+00	5.91E+00	7.61E-05	3.03E-03	4.13E-01
ENSRNOC	---	5.87E-01	1.00E+00	8.47E-01	-1.18E+00	3.30E+00	3.89E+00	1.72E-03	4.27E-02	4.16E-01
Plk4	Plk4	5.19E-01	1.00E+00	1.17E+00	1.17E+00	1.18E+01	1.01E+01	6.21E-05	2.55E-03	4.17E-01
Rrm2	Rrm2	5.98E-01	1.00E+00	1.16E+00	1.16E+00	6.00E+00	5.19E+00	6.01E-04	1.78E-02	4.19E-01

Cdca8	Cdca8	9.35E-01	1.00E+00	1.02E+00	1.02E+00	7.59E+00	7.43E+00	3.18E-04	1.06E-02	4.22E-01
ENSRNOC	---	7.49E-01	1.00E+00	1.04E+00	1.04E+00	2.12E+01	2.04E+01	1.10E-14	2.55E-12	4.22E-01
Bub1	Bub1	9.89E-01	1.00E+00	1.00E+00	1.00E+00	1.40E+01	1.40E+01	1.24E-09	1.42E-07	4.24E-01
Hmmr	Hmmr	6.52E-01	1.00E+00	1.11E+00	1.11E+00	1.16E+01	1.05E+01	5.07E-05	2.13E-03	4.27E-01
Zfp367	Zfp367	7.86E-01	1.00E+00	1.07E+00	1.07E+00	9.98E+00	9.38E+00	6.22E-05	2.55E-03	4.29E-01
Hjurp	Hjurp	8.77E-01	1.00E+00	1.03E+00	1.03E+00	7.26E+00	7.02E+00	1.72E-05	8.30E-04	4.31E-01
Espl1	Espl1	8.18E-01	1.00E+00	1.06E+00	1.06E+00	7.45E+00	7.04E+00	1.39E-04	5.04E-03	4.32E-01
ENSRNOC	---	6.93E-01	1.00E+00	9.59E-01	-1.04E+00	3.49E+01	3.64E+01	1.08E-17	3.52E-15	4.37E-01
Brca1	Brca1	7.75E-01	1.00E+00	9.53E-01	-1.05E+00	1.08E+01	1.13E+01	1.12E-07	9.41E-06	4.41E-01
Mcm3	Mcm3	5.26E-01	1.00E+00	1.10E+00	1.10E+00	3.60E+01	3.25E+01	9.28E-09	9.39E-07	4.41E-01
Col11a1	Col11a1	9.26E-01	1.00E+00	9.84E-01	-1.02E+00	1.17E+01	1.19E+01	1.72E-07	1.42E-05	4.45E-01
Dhrs3	Dhrs3	4.56E-01	1.00E+00	1.14E+00	1.14E+00	1.38E+01	1.21E+01	6.06E-07	4.47E-05	4.46E-01
H1f5	H1f5	2.67E-01	1.00E+00	1.18E+00	1.18E+00	2.88E+01	2.44E+01	3.61E-09	3.88E-07	4.48E-01
Tspan17	Tspan17	9.66E-01	1.00E+00	9.92E-01	-1.01E+00	1.04E+01	1.04E+01	2.61E-06	1.56E-04	4.55E-01
Knl1	Knl1	6.84E-01	1.00E+00	1.05E+00	1.05E+00	1.95E+01	1.86E+01	6.80E-14	1.46E-11	4.58E-01
Stil	Stil	9.89E-01	1.00E+00	1.00E+00	1.00E+00	7.51E+00	7.49E+00	2.83E-06	1.66E-04	4.61E-01
Aurka	Aurka	8.67E-01	1.00E+00	9.60E-01	-1.04E+00	4.22E+00	4.40E+00	6.09E-04	1.80E-02	4.62E-01
Kif15	Kif15	4.21E-01	1.00E+00	1.17E+00	1.17E+00	1.13E+01	9.60E+00	2.24E-05	1.04E-03	4.64E-01
Mad2l1	Mad2l1	8.17E-01	1.00E+00	1.06E+00	1.06E+00	5.77E+00	5.43E+00	1.53E-03	3.90E-02	4.66E-01
Plxna4	Plxna4	3.18E-01	1.00E+00	7.63E-01	-1.31E+00	4.17E+00	5.47E+00	1.91E-03	4.65E-02	4.67E-01
Dpep1	Dpep1	3.09E-01	1.00E+00	8.20E-01	-1.22E+00	1.27E+01	1.55E+01	1.96E-05	9.27E-04	4.72E-01
Dlgap5	Dlgap5	6.15E-01	1.00E+00	1.12E+00	1.12E+00	9.83E+00	8.77E+00	2.68E-04	9.00E-03	4.73E-01
Kcnk6	Kcnk6	5.82E-01	1.00E+00	1.13E+00	1.13E+00	8.69E+00	7.69E+00	3.26E-04	1.08E-02	4.83E-01
Vegfd	Vegfd	4.28E-01	1.00E+00	8.95E-01	-1.12E+00	1.32E+01	1.47E+01	1.27E-08	1.26E-06	4.84E-01
Fanci	Fanci	3.67E-01	1.00E+00	1.20E+00	1.20E+00	9.91E+00	8.27E+00	8.78E-05	3.42E-03	4.85E-01
Cdc20	Cdc20	4.79E-01	1.00E+00	1.12E+00	1.12E+00	1.06E+01	9.47E+00	4.94E-07	3.72E-05	4.85E-01
Dtl	Dtl	9.19E-01	1.00E+00	1.02E+00	1.02E+00	5.79E+00	5.65E+00	1.16E-03	3.08E-02	4.92E-01
Trip13	Trip13	9.96E-01	1.00E+00	9.99E-01	-1.00E+00	6.33E+00	6.33E+00	3.58E-04	1.17E-02	4.94E-01
Myo7a	Myo7a	8.22E-01	1.00E+00	1.04E+00	1.04E+00	1.44E+01	1.39E+01	3.60E-06	2.07E-04	4.95E-01
Fbxo5	Fbxo5	3.25E-01	1.00E+00	1.26E+00	1.26E+00	5.68E+00	4.50E+00	1.46E-03	3.73E-02	4.99E-01
ENSRNOC	---	6.80E-01	1.00E+00	9.55E-01	-1.05E+00	1.78E+01	1.86E+01	4.34E-13	8.09E-11	2.03E+00
Pde7b	Pde7b	4.76E-01	1.00E+00	8.65E-01	-1.16E+00	4.88E+00	5.64E+00	6.84E-05	2.74E-03	2.04E+00
Ccng1	Ccng1	7.60E-01	1.00E+00	1.02E+00	1.02E+00	2.00E+02	1.96E+02	3.91E-39	4.60E-36	2.04E+00
Btg2	Btg2	4.24E-01	1.00E+00	1.09E+00	1.09E+00	2.60E+01	2.38E+01	2.02E-13	3.92E-11	2.05E+00
Nqo1	Nqo1	8.34E-01	1.00E+00	1.02E+00	1.02E+00	2.66E+01	2.60E+01	1.59E-14	3.66E-12	2.07E+00
Slc20a1	Slc20a1	1.86E-01	1.00E+00	1.09E+00	1.09E+00	6.75E+01	6.21E+01	1.78E-39	2.23E-36	2.08E+00
Hmox1	Hmox1	5.17E-01	1.00E+00	9.48E-01	-1.06E+00	5.41E+01	5.71E+01	5.61E-24	3.41E-21	2.09E+00
AABR0700	AABR0700	9.96E-01	1.00E+00	1.00E+00	1.00E+00	7.59E+00	7.58E+00	2.58E-05	1.18E-03	2.10E+00
Pde4b	Pde4b	7.82E-01	1.00E+00	1.05E+00	1.05E+00	7.64E+00	7.29E+00	4.32E-07	3.32E-05	2.11E+00
Mt1	Mt1	1.47E-01	1.00E+00	1.16E+00	1.16E+00	5.41E+01	4.67E+01	8.24E-17	2.46E-14	2.12E+00
Chchd10	Chchd10	6.17E-01	1.00E+00	9.20E-01	-1.09E+00	8.43E+00	9.17E+00	2.10E-07	1.70E-05	2.14E+00
Inka2	Inka2	7.39E-01	1.00E+00	9.10E-01	-1.10E+00	1.85E+00	2.03E+00	1.91E-03	4.65E-02	2.15E+00
AC128848.	AC128848.	5.97E-01	1.00E+00	1.09E+00	1.09E+00	2.64E+01	2.43E+01	5.61E-08	4.96E-06	2.16E+00
Csf1	Csf1	8.00E-01	1.00E+00	9.90E-01	-1.01E+00	2.11E+02	2.13E+02	6.01E-103	3.77E-99	2.17E+00
Fas	Fas	9.63E-02	1.00E+00	1.24E+00	1.24E+00	7.78E+00	6.27E+00	9.17E-12	1.40E-09	2.17E+00
ENSRNOC	---	2.56E-01	1.00E+00	1.24E+00	1.24E+00	4.02E+01	3.23E+01	6.42E-06	3.47E-04	2.17E+00
Cd55	Cd55	7.98E-01	1.00E+00	1.03E+00	1.03E+00	2.21E+01	2.16E+01	6.24E-20	2.73E-17	2.18E+00
C4b	C4b	3.69E-01	1.00E+00	9.24E-01	-1.08E+00	2.23E+01	2.41E+01	2.13E-24	1.33E-21	2.19E+00
Lypd1	Lypd1	7.45E-01	1.00E+00	1.06E+00	1.06E+00	5.76E+00	5.42E+00	1.45E-06	9.20E-05	2.21E+00

Pde4d	Pde4d	8.89E-01	1.00E+00	9.79E-01	-1.02E+00	8.21E+00	8.38E+00	1.16E-09	1.34E-07	2.21E+00
Col7a1	Col7a1	1.18E-01	1.00E+00	7.32E-01	-1.37E+00	3.18E+00	4.35E+00	3.06E-06	1.78E-04	2.23E+00
Slc27a3	Slc27a3	2.24E-01	1.00E+00	1.15E+00	1.15E+00	1.13E+01	9.80E+00	2.22E-16	6.15E-14	2.27E+00
Slc19a2	Slc19a2	6.62E-01	1.00E+00	9.30E-01	-1.08E+00	6.30E+00	6.78E+00	1.57E-08	1.54E-06	2.27E+00
Abca1	Abca1	8.32E-01	1.00E+00	9.89E-01	-1.01E+00	1.19E+02	1.21E+02	3.55E-66	7.43E-63	2.29E+00
ENSRNOC	---	9.34E-01	1.00E+00	1.01E+00	1.01E+00	2.44E+02	2.41E+02	7.46E-12	1.17E-09	2.29E+00
Fdx1	Fdx1	3.42E-01	1.00E+00	1.14E+00	1.14E+00	1.18E+01	1.04E+01	1.99E-12	3.38E-10	2.30E+00
Aen	Aen	7.25E-01	1.00E+00	1.03E+00	1.03E+00	3.71E+01	3.60E+01	1.24E-31	1.17E-28	2.31E+00
Rgs2	Rgs2	9.82E-01	1.00E+00	1.00E+00	1.00E+00	4.79E+01	4.78E+01	1.58E-39	2.13E-36	2.37E+00
Ephx1	Ephx1	5.37E-01	1.00E+00	1.07E+00	1.07E+00	2.72E+01	2.54E+01	3.15E-19	1.29E-16	2.38E+00
Tp53inp1	Tp53inp1	6.20E-01	1.00E+00	1.06E+00	1.06E+00	1.50E+01	1.42E+01	4.27E-16	1.15E-13	2.40E+00
Lif	Lif	9.41E-01	1.00E+00	9.83E-01	-1.02E+00	3.83E+00	3.90E+00	1.23E-05	6.19E-04	2.45E+00
B3gnt7	B3gnt7	2.85E-01	1.00E+00	7.60E-01	-1.32E+00	2.62E+00	3.45E+00	5.39E-05	2.25E-03	2.46E+00
Cxcl1	Cxcl1	6.14E-01	1.00E+00	1.06E+00	1.06E+00	1.52E+01	1.43E+01	1.70E-17	5.43E-15	2.48E+00
Eda2r	Eda2r	6.21E-01	1.00E+00	9.45E-01	-1.06E+00	9.57E+00	1.01E+01	2.46E-20	1.13E-17	2.48E+00
Ptx3	Ptx3	3.99E-01	1.00E+00	1.07E+00	1.07E+00	3.15E+01	2.95E+01	1.17E-43	1.70E-40	2.62E+00
AABR0704	AABR0704	8.06E-01	1.00E+00	1.08E+00	1.08E+00	9.95E+00	9.25E+00	1.46E-04	5.23E-03	2.72E+00
Mdm2	Mdm2	4.20E-01	1.00E+00	1.06E+00	1.06E+00	5.81E+01	5.49E+01	5.71E-67	1.34E-63	2.93E+00
Bbc3	Bbc3	6.28E-01	1.00E+00	1.07E+00	1.07E+00	7.62E+00	7.14E+00	2.14E-21	1.04E-18	3.02E+00
Csf2rb	Csf2rb	9.34E-01	1.00E+00	9.72E-01	-1.03E+00	1.05E+00	1.08E+00	6.13E-05	2.53E-03	3.17E+00
Cd80	Cd80	2.19E-01	1.00E+00	1.34E+00	1.34E+00	3.27E+00	2.45E+00	4.64E-09	4.85E-07	3.36E+00
Abcb1b	Abcb1b	5.04E-01	1.00E+00	1.05E+00	1.05E+00	2.40E+01	2.29E+01	2.99E-101	1.41E-97	3.66E+00
Star	Star	1.35E-02	1.00E+00	1.15E+00	1.15E+00	9.80E+01	8.51E+01	2.53E-188	4.76E-184	4.36E+00
Cdkn1a	Cdkn1a	5.28E-01	1.00E+00	1.04E+00	1.04E+00	4.31E+01	4.12E+01	1.35E-155	1.27E-151	4.86E+00
Il6	Il6	8.28E-01	1.00E+00	9.27E-01	-1.08E+00	9.35E-01	1.01E+00	3.29E-08	3.10E-06	5.18E+00
Gdf15	Gdf15	2.33E-01	1.00E+00	1.37E+00	1.37E+00	1.96E+00	1.43E+00	2.02E-22	1.12E-19	9.17E+00

Fold chang	LSMean(G	LSMean(V	P-value (A	FDR step u	Ratio (AG5	Fold chang	LSMean(A	LSMean(Vehicle) (AG50 vs Vehic
-3.05E+01	4.81E-02	1.47E+00	6.40E-04	1.92E-02	7.97E-02	-1.26E+01	1.17E-01	1.47E+00
-1.70E+01	6.75E-02	1.15E+00	7.37E-01	9.43E-01	7.60E-01	-1.32E+00	8.75E-01	1.15E+00
-1.00E+01	7.61E-02	7.61E-01	2.72E-01	7.81E-01	5.22E-01	-1.92E+00	3.97E-01	7.61E-01
-7.87E+00	3.26E+00	2.57E+01	3.82E-39	5.53E-36	1.21E-01	-8.28E+00	3.10E+00	2.57E+01
-5.69E+00	8.29E-01	4.72E+00	9.53E-08	8.58E-06	1.85E-01	-5.40E+00	8.74E-01	4.72E+00
-4.99E+00	1.16E+00	5.80E+00	3.58E-05	1.66E-03	2.45E-01	-4.08E+00	1.42E+00	5.80E+00
-4.96E+00	2.90E-01	1.44E+00	3.09E-01	8.03E-01	6.14E-01	-1.63E+00	8.82E-01	1.44E+00
-4.94E+00	4.53E-01	2.24E+00	2.67E-04	9.41E-03	2.25E-01	-4.45E+00	5.03E-01	2.24E+00
-4.71E+00	7.91E-01	3.73E+00	1.40E-08	1.43E-06	1.91E-01	-5.23E+00	7.13E-01	3.73E+00
-4.71E+00	4.96E-01	2.33E+00	3.30E-03	7.10E-02	3.15E-01	-3.18E+00	7.35E-01	2.33E+00
-4.67E+00	6.78E-01	3.16E+00	2.33E-04	8.33E-03	2.40E-01	-4.17E+00	7.59E-01	3.16E+00
-4.44E+00	4.06E+00	1.80E+01	6.39E-26	4.81E-23	1.91E-01	-5.24E+00	3.44E+00	1.80E+01
-4.35E+00	1.41E+00	6.14E+00	2.71E-09	3.29E-07	2.78E-01	-3.60E+00	1.71E+00	6.14E+00
-4.07E+00	1.95E+00	7.92E+00	4.07E-09	4.64E-07	2.46E-01	-4.06E+00	1.95E+00	7.92E+00
-4.00E+00	2.16E+00	8.63E+00	1.40E-11	2.46E-09	2.68E-01	-3.73E+00	2.31E+00	8.63E+00
-3.91E+00	2.30E+00	9.02E+00	2.18E-14	5.48E-12	2.47E-01	-4.04E+00	2.23E+00	9.02E+00
-3.81E+00	7.52E-01	2.87E+00	7.54E-04	2.20E-02	2.77E-01	-3.61E+00	7.93E-01	2.87E+00
-3.78E+00	2.92E+00	1.10E+01	9.36E-09	1.01E-06	2.80E-01	-3.58E+00	3.08E+00	1.10E+01
-3.71E+00	4.51E-01	1.67E+00	2.28E-02	2.82E-01	4.17E-01	-2.40E+00	6.97E-01	1.67E+00
-3.66E+00	1.38E+00	5.05E+00	3.72E-05	1.71E-03	3.28E-01	-3.05E+00	1.65E+00	5.05E+00
-3.65E+00	1.38E+00	5.03E+00	2.86E-05	1.36E-03	2.75E-01	-3.64E+00	1.38E+00	5.03E+00
-3.63E+00	9.69E+00	3.52E+01	1.11E-22	6.96E-20	3.01E-01	-3.32E+00	1.06E+01	3.52E+01
-3.60E+00	6.52E+00	2.35E+01	5.08E-13	1.05E-10	2.60E-01	-3.85E+00	6.11E+00	2.35E+01
-3.59E+00	1.18E+00	4.25E+00	1.03E-04	4.10E-03	3.20E-01	-3.13E+00	1.36E+00	4.25E+00
-3.54E+00	2.04E+00	7.22E+00	5.00E-10	6.87E-08	2.38E-01	-4.20E+00	1.72E+00	7.22E+00
-3.53E+00	1.25E+00	4.40E+00	9.59E-07	6.76E-05	2.75E-01	-3.63E+00	1.21E+00	4.40E+00
-3.53E+00	7.35E-01	2.60E+00	9.68E-06	5.29E-04	2.58E-01	-3.88E+00	6.69E-01	2.60E+00
-3.50E+00	8.60E-01	3.01E+00	8.56E-05	3.48E-03	2.69E-01	-3.72E+00	8.09E-01	3.01E+00
-3.50E+00	2.05E+01	7.17E+01	1.28E-39	2.01E-36	2.69E-01	-3.71E+00	1.93E+01	7.17E+01
-3.46E+00	3.01E+00	1.04E+01	1.09E-09	1.43E-07	3.12E-01	-3.21E+00	3.24E+00	1.04E+01
-3.44E+00	8.04E-01	2.76E+00	5.13E-06	3.03E-04	2.42E-01	-4.14E+00	6.68E-01	2.76E+00
-3.44E+00	6.27E-01	2.16E+00	2.76E-03	6.23E-02	3.28E-01	-3.05E+00	7.07E-01	2.16E+00
-3.44E+00	2.01E+00	6.90E+00	1.36E-09	1.76E-07	2.77E-01	-3.62E+00	1.91E+00	6.90E+00
-3.43E+00	7.91E-01	2.71E+00	8.51E-04	2.44E-02	2.74E-01	-3.64E+00	7.43E-01	2.71E+00
-3.37E+00	1.22E+00	4.11E+00	1.37E-04	5.27E-03	3.14E-01	-3.18E+00	1.29E+00	4.11E+00
-3.35E+00	9.34E-01	3.13E+00	5.98E-07	4.45E-05	2.27E-01	-4.40E+00	7.12E-01	3.13E+00
-3.35E+00	3.36E+00	1.13E+01	3.30E-09	3.94E-07	3.31E-01	-3.02E+00	3.73E+00	1.13E+01
-3.34E+00	5.38E+00	1.80E+01	3.59E-18	1.50E-15	2.85E-01	-3.51E+00	5.13E+00	1.80E+01
-3.33E+00	1.17E+00	3.91E+00	4.79E-05	2.13E-03	3.34E-01	-2.99E+00	1.31E+00	3.91E+00
-3.31E+00	1.65E+00	5.45E+00	3.76E-07	2.96E-05	2.73E-01	-3.66E+00	1.49E+00	5.45E+00
-3.30E+00	2.17E+00	7.17E+00	3.87E-07	3.02E-05	3.02E-01	-3.32E+00	2.16E+00	7.17E+00
-3.29E+00	1.82E+00	6.00E+00	5.20E-09	5.73E-07	2.96E-01	-3.38E+00	1.78E+00	6.00E+00
-3.29E+00	8.12E+00	2.67E+01	1.18E-20	6.34E-18	3.50E-01	-2.86E+00	9.33E+00	2.67E+01
-3.27E+00	1.51E+00	4.93E+00	4.11E-07	3.18E-05	2.97E-01	-3.37E+00	1.47E+00	4.93E+00
-3.27E+00	1.88E+00	6.16E+00	1.77E-05	8.89E-04	2.87E-01	-3.49E+00	1.76E+00	6.16E+00
-3.22E+00	1.78E+00	5.72E+00	4.33E-05	1.96E-03	3.33E-01	-3.00E+00	1.91E+00	5.72E+00
-3.21E+00	1.24E+00	3.97E+00	6.88E-07	5.10E-05	2.47E-01	-4.04E+00	9.83E-01	3.97E+00

-3.21E+00	2.21E+00	7.10E+00	7.84E-07	5.75E-05	3.12E-01	-3.20E+00	2.22E+00	7.10E+00
-3.19E+00	1.77E+00	5.65E+00	9.45E-07	6.74E-05	3.10E-01	-3.23E+00	1.75E+00	5.65E+00
-3.19E+00	2.49E+00	7.95E+00	4.34E-05	1.96E-03	4.02E-01	-2.49E+00	3.20E+00	7.95E+00
-3.17E+00	2.38E+00	7.56E+00	1.56E-07	1.33E-05	3.00E-01	-3.33E+00	2.27E+00	7.56E+00
-3.16E+00	1.22E+00	3.85E+00	2.29E-06	1.47E-04	3.00E-01	-3.33E+00	1.16E+00	3.85E+00
-3.15E+00	3.11E+00	9.80E+00	2.06E-07	1.71E-05	3.11E-01	-3.22E+00	3.05E+00	9.80E+00
-3.07E+00	1.03E+01	3.17E+01	1.00E-30	9.96E-28	3.20E-01	-3.13E+00	1.01E+01	3.17E+01
-3.07E+00	3.95E+00	1.21E+01	5.88E-07	4.41E-05	4.53E-01	-2.21E+00	5.49E+00	1.21E+01
-3.07E+00	2.61E+00	7.99E+00	1.83E-03	4.53E-02	3.76E-01	-2.66E+00	3.00E+00	7.99E+00
-3.06E+00	8.14E-01	2.49E+00	1.45E-03	3.77E-02	3.85E-01	-2.60E+00	9.57E-01	2.49E+00
-3.05E+00	1.06E+00	3.23E+00	9.07E-03	1.50E-01	4.45E-01	-2.25E+00	1.44E+00	3.23E+00
-3.04E+00	1.19E+00	3.62E+00	9.39E-05	3.78E-03	2.82E-01	-3.54E+00	1.02E+00	3.62E+00
-3.02E+00	2.62E+00	7.91E+00	9.80E-08	8.73E-06	3.39E-01	-2.95E+00	2.68E+00	7.91E+00
-3.01E+00	1.73E+00	5.22E+00	4.47E-04	1.43E-02	4.70E-01	-2.13E+00	2.45E+00	5.22E+00
-3.01E+00	3.34E+00	1.01E+01	2.81E-07	2.24E-05	3.16E-01	-3.16E+00	3.19E+00	1.01E+01
-2.99E+00	6.18E+00	1.85E+01	5.60E-12	1.06E-09	3.35E-01	-2.98E+00	6.19E+00	1.85E+01
-2.98E+00	3.66E+00	1.09E+01	6.35E-09	6.95E-07	3.56E-01	-2.81E+00	3.88E+00	1.09E+01
-2.97E+00	1.52E+00	4.52E+00	8.85E-04	2.51E-02	4.50E-01	-2.22E+00	2.04E+00	4.52E+00
-2.93E+00	1.57E+00	4.61E+00	9.18E-04	2.59E-02	3.61E-01	-2.77E+00	1.66E+00	4.61E+00
-2.91E+00	7.13E+00	2.07E+01	4.91E-15	1.42E-12	3.50E-01	-2.85E+00	7.26E+00	2.07E+01
-2.82E+00	5.72E+00	1.61E+01	1.22E-10	1.83E-08	3.18E-01	-3.15E+00	5.12E+00	1.61E+01
-2.82E+00	6.04E+00	1.70E+01	1.08E-11	1.96E-09	3.41E-01	-2.93E+00	5.80E+00	1.70E+01
-2.80E+00	4.07E+01	1.14E+02	4.70E-16	1.47E-13	3.80E-01	-2.63E+00	4.33E+01	1.14E+02
-2.80E+00	2.77E+00	7.75E+00	1.73E-09	2.18E-07	3.14E-01	-3.19E+00	2.43E+00	7.75E+00
-2.78E+00	2.26E+00	6.29E+00	2.48E-03	5.74E-02	4.71E-01	-2.12E+00	2.96E+00	6.29E+00
-2.78E+00	1.06E+00	2.94E+00	7.88E-04	2.29E-02	3.74E-01	-2.68E+00	1.10E+00	2.94E+00
-2.77E+00	2.83E+00	7.82E+00	4.23E-06	2.55E-04	3.56E-01	-2.81E+00	2.79E+00	7.82E+00
-2.71E+00	1.40E+00	3.80E+00	4.49E-05	2.01E-03	3.57E-01	-2.80E+00	1.36E+00	3.80E+00
-2.70E+00	6.95E+00	1.88E+01	6.88E-18	2.81E-15	3.65E-01	-2.74E+00	6.86E+00	1.88E+01
-2.67E+00	2.24E+00	5.98E+00	1.94E-03	4.74E-02	4.26E-01	-2.35E+00	2.55E+00	5.98E+00
-2.63E+00	1.26E+00	3.32E+00	1.38E-03	3.63E-02	4.36E-01	-2.30E+00	1.45E+00	3.32E+00
-2.59E+00	1.01E+00	2.62E+00	2.58E-03	5.90E-02	4.13E-01	-2.42E+00	1.08E+00	2.62E+00
-2.58E+00	4.45E+00	1.15E+01	1.20E-07	1.05E-05	3.59E-01	-2.79E+00	4.11E+00	1.15E+01
-2.56E+00	1.18E+00	3.04E+00	1.62E-03	4.15E-02	3.90E-01	-2.56E+00	1.19E+00	3.04E+00
-2.56E+00	2.44E+00	6.26E+00	9.63E-09	1.04E-06	3.28E-01	-3.05E+00	2.05E+00	6.26E+00
-2.53E+00	1.81E+00	4.58E+00	1.87E-04	6.84E-03	4.59E-01	-2.18E+00	2.10E+00	4.58E+00
-2.53E+00	1.41E+00	3.56E+00	1.83E-03	4.53E-02	4.33E-01	-2.31E+00	1.54E+00	3.56E+00
-2.52E+00	2.88E+00	7.27E+00	1.65E-05	8.41E-04	4.63E-01	-2.16E+00	3.36E+00	7.27E+00
-2.49E+00	1.86E+00	4.64E+00	1.30E-04	5.05E-03	3.67E-01	-2.73E+00	1.70E+00	4.64E+00
-2.48E+00	2.45E+00	6.07E+00	1.32E-08	1.36E-06	2.95E-01	-3.39E+00	1.79E+00	6.07E+00
-2.46E+00	4.42E+00	1.09E+01	3.57E-09	4.23E-07	3.29E-01	-3.04E+00	3.58E+00	1.09E+01
-2.46E+00	5.40E+00	1.33E+01	2.13E-13	4.61E-11	3.99E-01	-2.51E+00	5.29E+00	1.33E+01
-2.45E+00	3.32E+00	8.14E+00	5.71E-05	2.46E-03	4.84E-01	-2.07E+00	3.94E+00	8.14E+00
-2.44E+00	7.54E+00	1.84E+01	2.30E-16	7.74E-14	3.51E-01	-2.85E+00	6.46E+00	1.84E+01
-2.43E+00	2.51E+00	6.09E+00	1.98E-06	1.29E-04	3.95E-01	-2.53E+00	2.41E+00	6.09E+00
-2.42E+00	2.44E+00	5.91E+00	4.40E-07	3.37E-05	3.18E-01	-3.14E+00	1.88E+00	5.91E+00
-2.41E+00	1.62E+00	3.89E+00	6.57E-02	4.96E-01	6.01E-01	-1.66E+00	2.34E+00	3.89E+00
-2.40E+00	4.23E+00	1.01E+01	1.88E-05	9.37E-04	3.92E-01	-2.55E+00	3.98E+00	1.01E+01
-2.39E+00	2.17E+00	5.19E+00	1.37E-04	5.27E-03	3.79E-01	-2.64E+00	1.97E+00	5.19E+00

-2.37E+00	3.14E+00	7.43E+00	6.41E-04	1.92E-02	4.42E-01	-2.26E+00	3.28E+00	7.43E+00
-2.37E+00	8.60E+00	2.04E+01	1.75E-13	3.82E-11	4.40E-01	-2.27E+00	8.98E+00	2.04E+01
-2.36E+00	5.94E+00	1.40E+01	2.02E-09	2.51E-07	4.30E-01	-2.33E+00	6.01E+00	1.40E+01
-2.34E+00	4.47E+00	1.05E+01	3.58E-06	2.22E-04	3.77E-01	-2.65E+00	3.94E+00	1.05E+01
-2.33E+00	4.02E+00	9.38E+00	5.52E-05	2.39E-03	4.26E-01	-2.35E+00	3.99E+00	9.38E+00
-2.32E+00	3.03E+00	7.02E+00	4.32E-06	2.58E-04	4.06E-01	-2.46E+00	2.85E+00	7.02E+00
-2.31E+00	3.05E+00	7.04E+00	1.12E-05	5.95E-04	3.79E-01	-2.64E+00	2.67E+00	7.04E+00
-2.29E+00	1.59E+01	3.64E+01	5.20E-21	2.89E-18	4.02E-01	-2.49E+00	1.46E+01	3.64E+01
-2.27E+00	4.98E+00	1.13E+01	2.12E-05	1.05E-03	5.21E-01	-1.92E+00	5.89E+00	1.13E+01
-2.27E+00	1.44E+01	3.25E+01	5.21E-11	8.45E-09	3.91E-01	-2.56E+00	1.27E+01	3.25E+01
-2.25E+00	5.30E+00	1.19E+01	8.85E-06	4.86E-04	5.04E-01	-1.98E+00	6.01E+00	1.19E+01
-2.24E+00	5.40E+00	1.21E+01	3.77E-13	7.98E-11	3.03E-01	-3.30E+00	3.66E+00	1.21E+01
-2.23E+00	1.09E+01	2.44E+01	3.71E-09	4.36E-07	4.48E-01	-2.23E+00	1.09E+01	2.44E+01
-2.20E+00	4.76E+00	1.04E+01	4.36E-04	1.40E-02	5.58E-01	-1.79E+00	5.82E+00	1.04E+01
-2.18E+00	8.52E+00	1.86E+01	9.84E-15	2.73E-12	4.46E-01	-2.24E+00	8.29E+00	1.86E+01
-2.17E+00	3.46E+00	7.49E+00	7.97E-03	1.37E-01	6.50E-01	-1.54E+00	4.87E+00	7.49E+00
-2.16E+00	2.03E+00	4.40E+00	5.94E-04	1.81E-02	4.61E-01	-2.17E+00	2.03E+00	4.40E+00
-2.15E+00	4.46E+00	9.60E+00	1.85E-06	1.22E-04	4.20E-01	-2.38E+00	4.04E+00	9.60E+00
-2.14E+00	2.53E+00	5.43E+00	4.00E-03	8.25E-02	5.01E-01	-2.00E+00	2.72E+00	5.43E+00
-2.14E+00	2.55E+00	5.47E+00	2.55E-03	5.86E-02	4.78E-01	-2.09E+00	2.61E+00	5.47E+00
-2.12E+00	7.30E+00	1.55E+01	1.84E-05	9.21E-04	4.71E-01	-2.12E+00	7.28E+00	1.55E+01
-2.12E+00	4.15E+00	8.77E+00	8.02E-05	3.28E-03	4.44E-01	-2.25E+00	3.89E+00	8.77E+00
-2.07E+00	3.71E+00	7.69E+00	6.84E-03	1.24E-01	5.80E-01	-1.72E+00	4.46E+00	7.69E+00
-2.06E+00	7.12E+00	1.47E+01	3.74E-09	4.38E-07	4.72E-01	-2.12E+00	6.94E+00	1.47E+01
-2.06E+00	4.01E+00	8.27E+00	5.73E-03	1.09E-01	6.03E-01	-1.66E+00	4.99E+00	8.27E+00
-2.06E+00	4.60E+00	9.47E+00	2.52E-08	2.47E-06	4.47E-01	-2.23E+00	4.24E+00	9.47E+00
-2.03E+00	2.78E+00	5.65E+00	1.06E-03	2.90E-02	4.89E-01	-2.04E+00	2.77E+00	5.65E+00
-2.02E+00	3.13E+00	6.33E+00	1.45E-05	7.47E-04	4.22E-01	-2.37E+00	2.67E+00	6.33E+00
-2.02E+00	6.88E+00	1.39E+01	1.44E-06	9.79E-05	4.81E-01	-2.08E+00	6.69E+00	1.39E+01
-2.00E+00	2.24E+00	4.50E+00	1.82E-03	4.53E-02	5.07E-01	-1.97E+00	2.28E+00	4.50E+00
2.03E+00	3.78E+01	1.86E+01	1.64E-04	6.14E-03	1.45E+00	1.45E+00	2.69E+01	1.86E+01
2.04E+00	1.15E+01	5.64E+00	1.41E-03	3.69E-02	1.77E+00	1.77E+00	9.98E+00	5.64E+00
2.04E+00	4.01E+02	1.96E+02	4.86E-30	4.36E-27	1.86E+00	1.86E+00	3.65E+02	1.96E+02
2.05E+00	4.88E+01	2.38E+01	2.46E-02	2.95E-01	1.25E+00	1.25E+00	2.97E+01	2.38E+01
2.07E+00	5.40E+01	2.60E+01	9.70E-23	6.30E-20	2.54E+00	2.54E+00	6.60E+01	2.60E+01
2.08E+00	1.29E+02	6.21E+01	4.10E-33	5.14E-30	1.95E+00	1.95E+00	1.21E+02	6.21E+01
2.09E+00	1.20E+02	5.71E+01	1.37E-18	6.00E-16	1.91E+00	1.91E+00	1.09E+02	5.71E+01
2.10E+00	1.59E+01	7.58E+00	1.52E-06	1.02E-04	2.33E+00	2.33E+00	1.76E+01	7.58E+00
2.11E+00	1.54E+01	7.29E+00	2.78E-07	2.23E-05	2.14E+00	2.14E+00	1.56E+01	7.29E+00
2.12E+00	9.88E+01	4.67E+01	1.99E-11	3.44E-09	1.83E+00	1.83E+00	8.54E+01	4.67E+01
2.14E+00	1.96E+01	9.17E+00	4.04E-06	2.46E-04	1.97E+00	1.97E+00	1.81E+01	9.17E+00
2.15E+00	4.37E+00	2.03E+00	1.44E-02	2.07E-01	1.83E+00	1.83E+00	3.73E+00	2.03E+00
2.16E+00	5.23E+01	2.43E+01	3.41E-07	2.70E-05	2.06E+00	2.06E+00	4.99E+01	2.43E+01
2.17E+00	4.63E+02	2.13E+02	1.01E-98	6.33E-95	2.14E+00	2.14E+00	4.56E+02	2.13E+02
2.17E+00	1.36E+01	6.27E+00	9.34E-10	1.26E-07	2.01E+00	2.01E+00	1.26E+01	6.27E+00
2.17E+00	7.02E+01	3.23E+01	2.77E-08	2.70E-06	2.60E+00	2.60E+00	8.39E+01	3.23E+01
2.18E+00	4.71E+01	2.16E+01	2.77E-17	1.06E-14	2.06E+00	2.06E+00	4.44E+01	2.16E+01
2.19E+00	5.28E+01	2.41E+01	1.04E-16	3.70E-14	1.90E+00	1.90E+00	4.57E+01	2.41E+01
2.21E+00	1.20E+01	5.42E+00	7.63E-05	3.14E-03	1.92E+00	1.92E+00	1.04E+01	5.42E+00

2.21E+00	1.86E+01	8.38E+00	2.74E-12	5.37E-10	2.49E+00	2.49E+00	2.08E+01	8.38E+00
2.23E+00	9.68E+00	4.35E+00	4.99E-05	2.20E-03	2.01E+00	2.01E+00	8.73E+00	4.35E+00
2.27E+00	2.23E+01	9.80E+00	7.96E-14	1.81E-11	2.11E+00	2.11E+00	2.07E+01	9.80E+00
2.27E+00	1.54E+01	6.78E+00	2.56E-05	1.23E-03	1.85E+00	1.85E+00	1.25E+01	6.78E+00
2.29E+00	2.76E+02	1.21E+02	6.70E-69	1.58E-65	2.33E+00	2.33E+00	2.81E+02	1.21E+02
2.29E+00	5.53E+02	2.41E+02	2.45E-08	2.41E-06	1.97E+00	1.97E+00	4.74E+02	2.41E+02
2.30E+00	2.38E+01	1.04E+01	1.63E-07	1.38E-05	1.86E+00	1.86E+00	1.93E+01	1.04E+01
2.31E+00	8.31E+01	3.60E+01	4.76E-22	2.80E-19	2.00E+00	2.00E+00	7.19E+01	3.60E+01
2.37E+00	1.13E+02	4.78E+01	1.91E-18	8.19E-16	1.78E+00	1.78E+00	8.50E+01	4.78E+01
2.38E+00	6.04E+01	2.54E+01	3.04E-19	1.43E-16	2.38E+00	2.38E+00	6.04E+01	2.54E+01
2.40E+00	3.39E+01	1.42E+01	4.58E-11	7.50E-09	2.03E+00	2.03E+00	2.88E+01	1.42E+01
2.45E+00	9.53E+00	3.90E+00	3.93E-03	8.17E-02	1.81E+00	1.81E+00	7.05E+00	3.90E+00
2.46E+00	8.47E+00	3.45E+00	5.14E-02	4.40E-01	1.55E+00	1.55E+00	5.33E+00	3.45E+00
2.48E+00	3.54E+01	1.43E+01	2.72E-06	1.71E-04	1.65E+00	1.65E+00	2.36E+01	1.43E+01
2.48E+00	2.51E+01	1.01E+01	5.03E-26	3.95E-23	2.82E+00	2.82E+00	2.85E+01	1.01E+01
2.62E+00	7.72E+01	2.95E+01	9.43E-32	1.05E-28	2.26E+00	2.26E+00	6.67E+01	2.95E+01
2.72E+00	2.52E+01	9.25E+00	3.39E-03	7.26E-02	2.17E+00	2.17E+00	2.00E+01	9.25E+00
2.93E+00	1.61E+02	5.49E+01	1.43E-30	1.35E-27	2.05E+00	2.05E+00	1.13E+02	5.49E+01
3.02E+00	2.15E+01	7.14E+00	6.86E-06	3.94E-04	1.70E+00	1.70E+00	1.21E+01	7.14E+00
3.17E+00	3.42E+00	1.08E+00	1.12E-03	3.03E-02	2.56E+00	2.56E+00	2.77E+00	1.08E+00
3.36E+00	8.22E+00	2.45E+00	3.24E-09	3.89E-07	3.40E+00	3.40E+00	8.32E+00	2.45E+00
3.66E+00	8.37E+01	2.29E+01	3.69E-113	3.48E-109	3.94E+00	3.94E+00	9.01E+01	2.29E+01
4.36E+00	3.71E+02	8.51E+01	3.91E-179	7.37E-175	4.21E+00	4.21E+00	3.58E+02	8.51E+01
4.86E+00	2.00E+02	4.12E+01	8.58E-94	4.04E-90	3.40E+00	3.40E+00	1.40E+02	4.12E+01
5.18E+00	5.22E+00	1.01E+00	8.36E-04	2.40E-02	2.73E+00	2.73E+00	2.75E+00	1.01E+00
9.17E+00	1.31E+01	1.43E+00	8.82E-15	2.48E-12	5.88E+00	5.88E+00	8.41E+00	1.43E+00
